# Sirolimus-eluting airway stent reduces profibrotic Th17 cells and inhibits laryngotracheal stenosis

**DOI:** 10.1172/jci.insight.158456

**Published:** 2023-06-08

**Authors:** Kevin M. Motz, Ioan A. Lina, Idris Samad, Michael K. Murphy, Madhavi Duvvuri, Ruth J. Davis, Alexander Gelbard, Liam Chung, Yee Chan-Li, Samuel Collins, Jonathan D. Powell, Jennifer H. Elisseeff, Maureen R. Horton, Alexander T. Hillel

**Affiliations:** 1 Department of Otolaryngology Head and Neck Surgery, Johns Hopkins School of Medicine, Baltimore, Maryland, USA.; 2 Department of Otolaryngology, Stanford University School of Medicine, Stanford, California, USA.; 3 Department of Otolaryngology, State University of New York, Upstate Medical University, Syracuse, New York, USA.; 4 Department of Radiology, University of California, San Francisco, San Francisco, California, USA.; 5 Department of Otolaryngology Head and Neck Surgery, Vanderbilt University Medical Center, Nashville, Tennessee, USA.; 6 Translational Tissue Engineering Center, Wilmer Eye Institute, and Department of Biomedical Engineering,; 7 Department of Oncology, and; 8 Department of Medicine, Johns Hopkins University School of Medicine, Baltimore, Maryland, USA.

**Keywords:** Immunology, Pulmonology, Adaptive immunity, Fibrosis, T cells

## Abstract

Laryngotracheal stenosis (LTS) is pathologic fibrotic narrowing of the larynx and trachea characterized by hypermetabolic fibroblasts and CD4^+^ T cell–mediated inflammation. However, the role of CD4^+^ T cells in promoting LTS fibrosis is unknown. The mTOR signaling pathways have been shown to regulate the T cell phenotype. Here we investigated the influence of mTOR signaling in CD4^+^ T cells on LTS pathogenesis. In this study, human LTS specimens revealed a higher population of CD4^+^ T cells expressing the activated isoform of mTOR. In a murine LTS model, targeting mTOR with systemic sirolimus and a sirolimus-eluting airway stent reduced fibrosis and Th17 cells. Selective deletion of mTOR in CD4^+^ cells reduced Th17 cells and attenuated fibrosis, demonstrating CD4^+^ T cells’ pathologic role in LTS. Multispectral immunofluorescence of human LTS revealed increased Th17 cells. In vitro, Th17 cells increased collagen-1 production by LTS fibroblasts, which was prevented with sirolimus pretreatment of Th17 cells. Collectively, mTOR signaling drove pathologic CD4^+^ T cell phenotypes in LTS, and targeting mTOR with sirolimus was effective at treating LTS through inhibition of profibrotic Th17 cells. Finally, sirolimus may be delivered locally with a drug-eluting stent, transforming clinical therapy for LTS.

## Introduction

Iatrogenic laryngotracheal stenosis (LTS) is a consequence of prolonged endotracheal intubation or a tracheostomy tube and is characterized by narrowing of the subglottic and tracheal airway by fibrosis. The resultant scar tissue causes a fixed extrathoracic obstruction and associated increased airway resistance that lead to dyspnea and potential progression to respiratory failure and death (1–3). LTS affects up to 11% of patients after prolonged intubation (4). Accordingly, risk factors for developing LTS include increased intubation duration, elevated endotracheal tube cuff pressure, or large endotracheal tube size. At present, the SARS-CoV-2 coronavirus pandemic is resulting in an increasing number of intubations from respiratory failure, with a corresponding increase in incidence of LTS (5). Underlying comorbidities such as diabetes mellitus, obesity, and cardiovascular disease are also known risk factors (1, 6–10). Other etiologies of LTS exist, including autoimmune and idiopathic etiologies, but the focus of this work is on iatrogenic LTS. Currently, the management of LTS is exclusively procedural, with the goal of increasing the airway diameter (11). However, this requires serial surgical procedures or a tracheostomy to bypass the level of obstruction, making alternate therapies highly desirable.

LTS was previously considered an irreversible disease, typified by overactive fibroblasts and excessive collagen 1 deposition (12). More recent studies demonstrated that the unregulated tissue remodeling seen in LTS is influenced by inflammation (13–16). Fibrosis is reduced in SCID mice (13), and in both murine and human studies, a T cell infiltrate not only precedes the development of fibrosis but also persists in patients with LTS compared with those without (16). Specifically, specimens from patients with LTS harbor a higher density of CD4^+^ T lymphocytes and show greater expression of Th2 and Th17 cytokines (14–16). Although the causal role of CD4^+^ T cell–mediated inflammation in LTS has not been established, these findings suggest that leveraging medical therapies that target cellular immunity could offer a new therapeutic option for LTS (13, 14, 16).

Sirolimus is an inhibitor of mTOR; it is known to curtail inflammation and associated downstream fibrosis. Specifically, mTOR inhibition blocks cell cycle progression during T cell activation and limits T cell differentiation through its subunits mTORC1 and mTORC2 (17). Sirolimus has been used therapeutically in CD4^+^ T cell–mediated fibrotic states, including idiopathic pulmonary fibrosis and renal interstitial fibrosis (18–20), making it a logical choice for testing as a potential therapeutic in LTS. In a small cohort of 4 patients, systemic sirolimus reduced LTS severity by reducing the number of necessary surgical procedures (21). In this study we demonstrated that systemic sirolimus suppresses CD4^+^ T cell–mediated inflammation, attenuates fibrosis, and increases survival in a mouse model of LTS. Subsequent in vivo mechanistic investigations showed that selective deletion of mTOR in CD4^+^ T cells suppresses the Th17 cell population, reduces fibrosis, and increases survival, proving a causal relationship between CD4^+^ T cell–mediated inflammation and LTS development. Finally, we demonstrated that our previously described proof-of-concept sirolimus-eluting airway stent (SEAS) elicited an antifibrotic effect and increased in vivo survival similar to that of systemic sirolimus (22). We demonstrated that T cell–mediated inflammation drives LTS development, systemic mTOR inhibition suppresses Th17 differentiation and attenuates fibrosis, and local delivery of sirolimus is equivalent to systemic treatment.

## Results

### LTS is an obstructive fibrotic disease with increased CD4^+^ T cells colocalizing p-mTOR.

**Figure 1:**
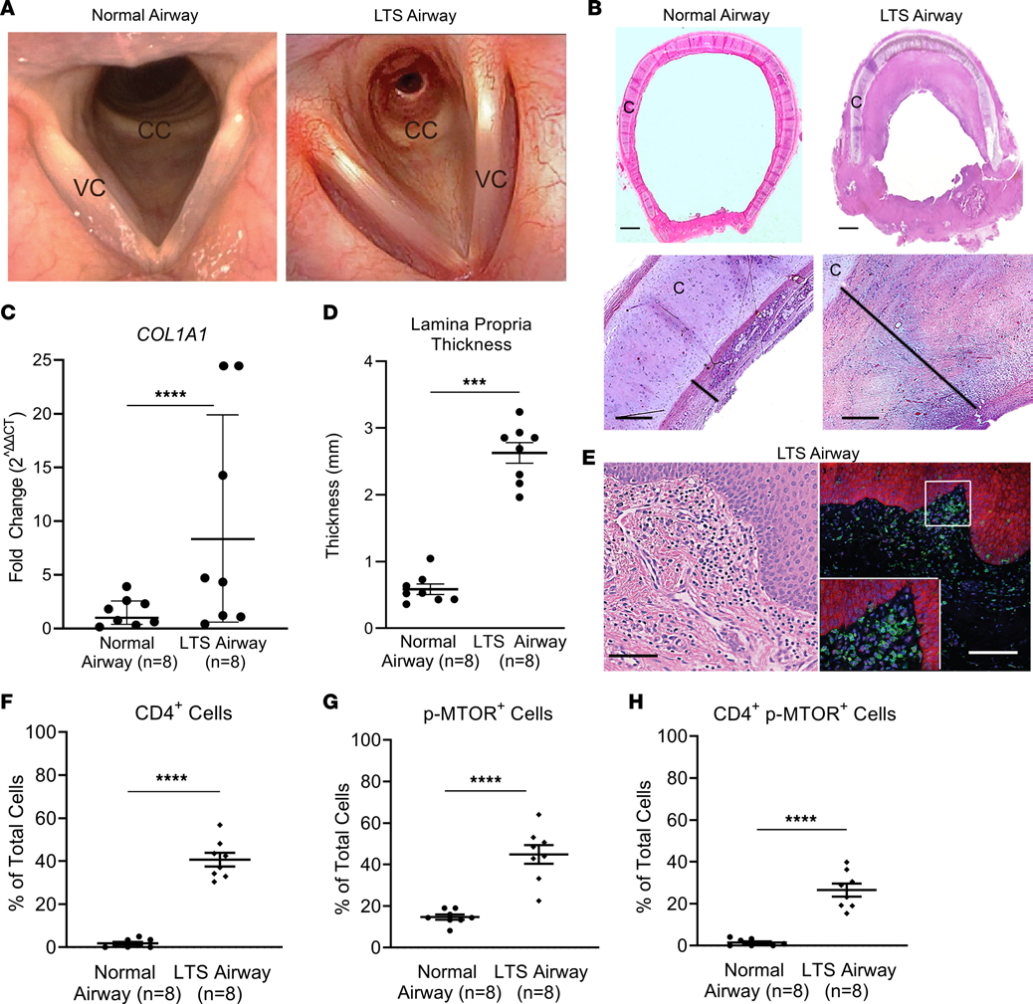
LTS is a fibrotic extrathoracic obstructive airway disease. (**A**) High-resolution endoscopic image of the proximal trachea in a normal human trachea (normal airway) compared with a patient with LTS (LTS airway), showing extensive narrowing of the airway. CC, cricoid cartilage; VC, vocal cord. (**B**) Photomicrograph (scale bar: 2 mm) and high-power (×20) microscopic images (scale bar: 200 μm) of H&E-stained sections from normal airway and an LTS airway. C, cartilage. (**C**) Quantitative analysis demonstrated increased LP thickness in human LTS specimens (2625.3 ± 435.7 μm, *n* = 8) compared with normal control specimens (584.4 ± 220.1 μm, *n* = 8). (**D**) *COL1A1* expression was greater in the LTS airway (8.33 ± 12.76) than in the normal trachea (0.99 ± 0.49). (**E**) Representative H&E (scale bar: 50 μm) and immunofluorescent staining (scale bar: 100 μm) for the cell surface marker CD4 and the activated form of the kinase mTOR (p-mTOR) shows human LTS has dense subepithelial CD4^+^ T cells (green surface fluorochrome) coexpressing p-mTOR (red intracellular fluorochrome). Quantitative analysis demonstrated greater density of (**F**) CD4^+^ cells in the LTS airway (40.69 ± 3.13) than in the normal airway (1.81 ± 0.671), (**G**) p-mTOR^+^ (44.85 ± 4.47 versus 14.70 ± 1.24) and (**H**) CD4^+^ p-mTOR^+^ (26.53 ± 3.10 versus 1.44 ± 0.568) cells in human LTS compared with normal control specimens. Data are presented as individual data points with error bars reflecting the mean ± SEM. Gene expression data are represented as average fold change (2^ΔΔCT^) and SEM. A Student’s *t* test was used to determine *P* values for comparisons. *P < 0.05, **P < 0.01, ***P < 0.001, ****P < 0.0001.

Endoscopic images demonstrated luminal narrowing of the subglottis and trachea in a patient with LTS compared with a normal subglottic airway in a healthy individual acting as a control (Figure 1A). Histopathologic assessment at ×10 and ×40 revealed excessive extracellular matrix deposition causing a thickened lamina propria (LP), the area between the cartilaginous superstructure and the luminal epithelium in LTS tracheas compared with normal tracheas (Figure 1B). Biopsies of the diseased LP in human LTS demonstrated greater collagen-1 (*COL1A1*) gene expression compared with healthy control tissue (Figure 1C). Measurements of the LP demonstrated greater thickness in human LTS specimens compared with control specimens (584.4 ± 220.1 μm versus 2625.3 ± 435.7; *P* < 0.001) (Figure 1D). Immunofluorescence (IF) for CD4 and the activated mTOR isoform, phosphorylated mTOR (p-mTOR), was performed on human LTS specimens (Figure 1E) and normal control specimens. Quantification of cells staining for CD4 and p-mTOR revealed an increase in CD4^+^ T cells (Figure 1F) and cells expressing p-mTOR (Figure 1G) in LTS specimens. Additionally, LTS specimens had an increase in cells expressing both CD4 and p-mTOR compared with normal control specimens (25.78% ± 8.2 versus 1.51% ±0.5, *P* < 0.0001) (Figure 1H). FACS analysis of human LTS specimens reveals that 51.23% of p-mTOR^+^ CD4^+^ T cells also express IL-17A, and 1.38% and 30.73% express IL-4 and IFN-γ, respectively. In addition, TH2 cells represented 1.07% of the overall CD4^+^ T cell population (Supplemental Figure 1; supplemental material is available online with this article; https://doi.org/10.1172/jci.insight.158456DS1).

### Inhibition of mTOR with sirolimus attenuates the development of LTS and suppresses the Th17 cell population.

**Figure 2:**
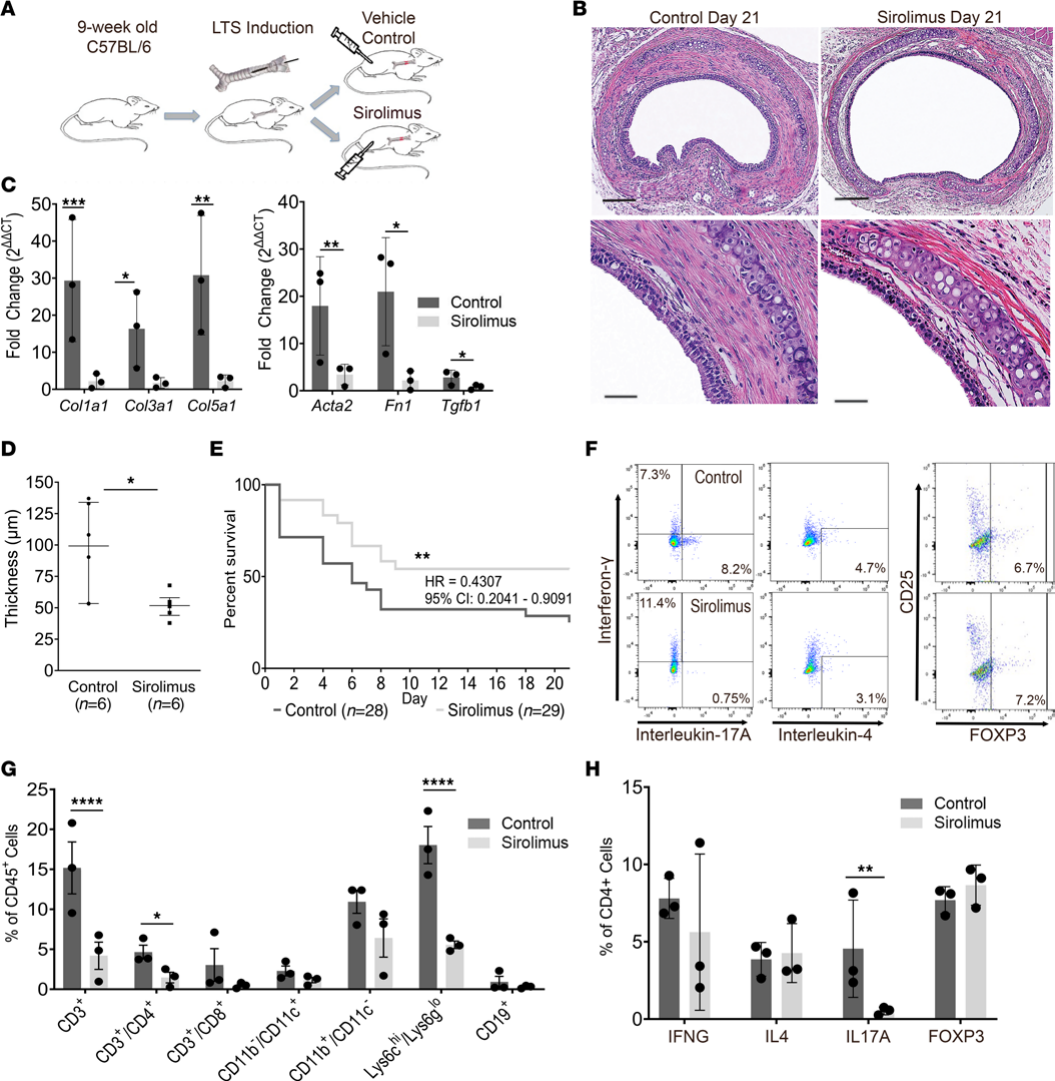
Systemic sirolimus reduced CD4^+^ T cell inflammation, attenuated fibrosis, and improved survival in LTS mice. Using a bleomycin-induced murine LTS model, 9-week-old C57BL/6 mice were treated with systemic sirolimus or vehicle control. (**A**) Experimental design. (**B**) Representative histology (original magnification, ×10, top; ×40, bottom) of LTS-induced murine tracheas at day 21. Scale bars: 200 μm (×10); 50 μm (×40). (**C**) Quantitative real-time PCR revealed reduced *Col1a1* (2.18 ± 1.98 vs 29.34 ± 16.47), *Col3a1* (1.75 ± 1.40 vs 16.34 ± 10.29), *Col5a1* (2.25 ± 1.63 vs 30.80 ± 16.11), *Acta2* (3.42 ± 2.17 vs 17.96 ± 10.41), *Fn1* (2.15 ± 2.84 vs 20.96 ± 11.46), and *Tgfb1* (0.78 ± 0.51 vs 2.82 ± 1.53) at day 7 in i.p. sirolimus-treated (*n* = 3) mice compared with controls (*n* = 3). (**D**) Tracheal lamina propria thickness (μm) at day 21 was reduced in i.p. sirolimus-treated mice (51.55 ± 4.056, *n* = 6) compared with controls (95.85 ± 15.13, *n* = 6). (**E**) 21-day Kaplan-Meier survival curve of LTS mice treated with i.p. sirolimus (gray line, *n* = 28) versus i.p. vehicle control (black line, *n* = 29) (HR, 2.427; 95% CI, 1.169–5.040; *P* = 0.0174). (**F**) Representative flow cytometry plots demonstrating CD4^+^ T cell phenotype. (**G**) Analysis of immune cell populations revealed reduced CD3^+^ (10.98 ± 3.67), CD4^+^ (3.19 ± 1.09), CD11b^+^/CD11^–^ (4.527 ±2.79), and Lys6c^hi^/Lys6g^lo^ (12.47 ± 2.37) cells in tracheas of i.p. sirolimus-treated mice. (**H**) i.p. sirolimus-treated mice (*n* = 3) demonstrated reduced Th17 cells (4.02 ± 1.82). Flow cytometry data are presented as mean percentage reduction ± SEM. A Mann-Whitney *U* test was used for comparative analysis of LP thickness presented as mean ± SEM. Survival differences were determined using a Mantel-Cox log-rank analysis and presented as HR with 95% CI. A 2-way ANOVA assessed differences in immune cell populations. An unpaired *t* test comparing ΔΔCT values was used to assess gene expression change, presented as fold change (2^ΔΔCT^) and SEM. *P < 0.05, **P < 0.01, ***P < 0.001, ****P < 0.0001.

Using an in vivo bleomycin-induced model of LTS (23) (Supplemental Figure 2), we investigated the antifibrotic and anti-inflammatory effects of the mTOR inhibitor sirolimus. LTS was induced in the proximal trachea of 10-week-old C57BL6 mice. LTS-induced mice were treated with an i.p. delivery of sirolimus (4 mg/kg) or a vehicle control (Figure 2A) every 48 hours. Histopathologic examination of the murine trachea at day 21 revealed a narrower, nonfibrotic LP in sirolimus-treated mice compared with progressive stenosis in vehicle controls (Figure 2B). Quantitative real-time PCR analysis of fibrosis-related gene expression revealed a reduction in collagen-1 (*Col1a1*), collagen-3 (*Col3a1*), collagen-5 (*Col5a1*), α smooth muscle actin (*Acta2*), *Tgfb1*, and fibronectin (*Fn1*) gene expression at day 7 in sirolimus-treated mice (Figure 2C). Nonsignificant decreases in these markers were observed at days 14 and 21 (Supplemental Figure 3). Quantitative assessment of LP thickness revealed a thinner LP in the i.p. sirolimus-treated mice compared with controls at days 7, 14 (Supplemental Figure 4), and 21 (51.6 ± 9.9 versus 95.9 ± 37.1 μm; *P* = 0.026; Figure 2D). Qualitatively, ACTA2 was reduced in the LP of sirolimus-treated mice at day 7 as assessed by IF (Supplemental Figure 5, A and B). A 21-day Kaplan-Meier estimate of survival demonstrated a survival benefit for the group receiving i.p. sirolimus (HR = 0.4307; 95% CI, 0.2041–0.9091; Figure 2E).

To assess the impact of sirolimus on the tracheal immune environment, we used flow cytometry to compare inflammatory cells in the trachea of sirolimus-treated LTS mice to controls. FACS analysis was performed to assess immune cell infiltrate and CD4^+^ T cell phenotype (Figure 2F). Sirolimus treatment resulted in a global reduction in inflammatory cells, including significant reductions in the CD3, CD4, C11b/CD11^–^, and Lys6c^hi^/Lys6g^lo^ cell populations (Figure 2G). CD4^+^ T cell immunophenotype was assessed by intracellular staining for IFN-γ (Th1), IL-4 (Th2), IL-17A (Th17), and FOXP3 (T-regulatory), which revealed a reduction in the population of CD4^+^ cells that colocalize with IL-17A in the LP of i.p. sirolimus-treated LTS mice (*P* = 0.02; 2-way ANOVA) (Figure 2H; gating strategy demonstrated in Supplemental Figure 6). No changes were observed in the Th1, Th2, or T-regulatory populations. Additionally, no changes in the population of δγ T cells were observed with i.p. sirolimus (Supplemental Figure 7). These findings are consistent with the recognized role of mTOR signaling in CD4^+^ T cell phenotype determination (17).

### CD4-specific mTOR inhibition attenuates fibrosis in LTS and suppresses Th1 and Th17 inflammation.

To evaluate how mTOR inhibition in CD4^+^ T cells influences the development of LTS, a strain of mice expressing a CD4-Cre transgene and carrying a floxed *Mtor* allele were used to inactivate mTOR in CD4-expressing cells (17). Genotype was confirmed via PCR (Supplemental Figure 8).

**Figure 3:**
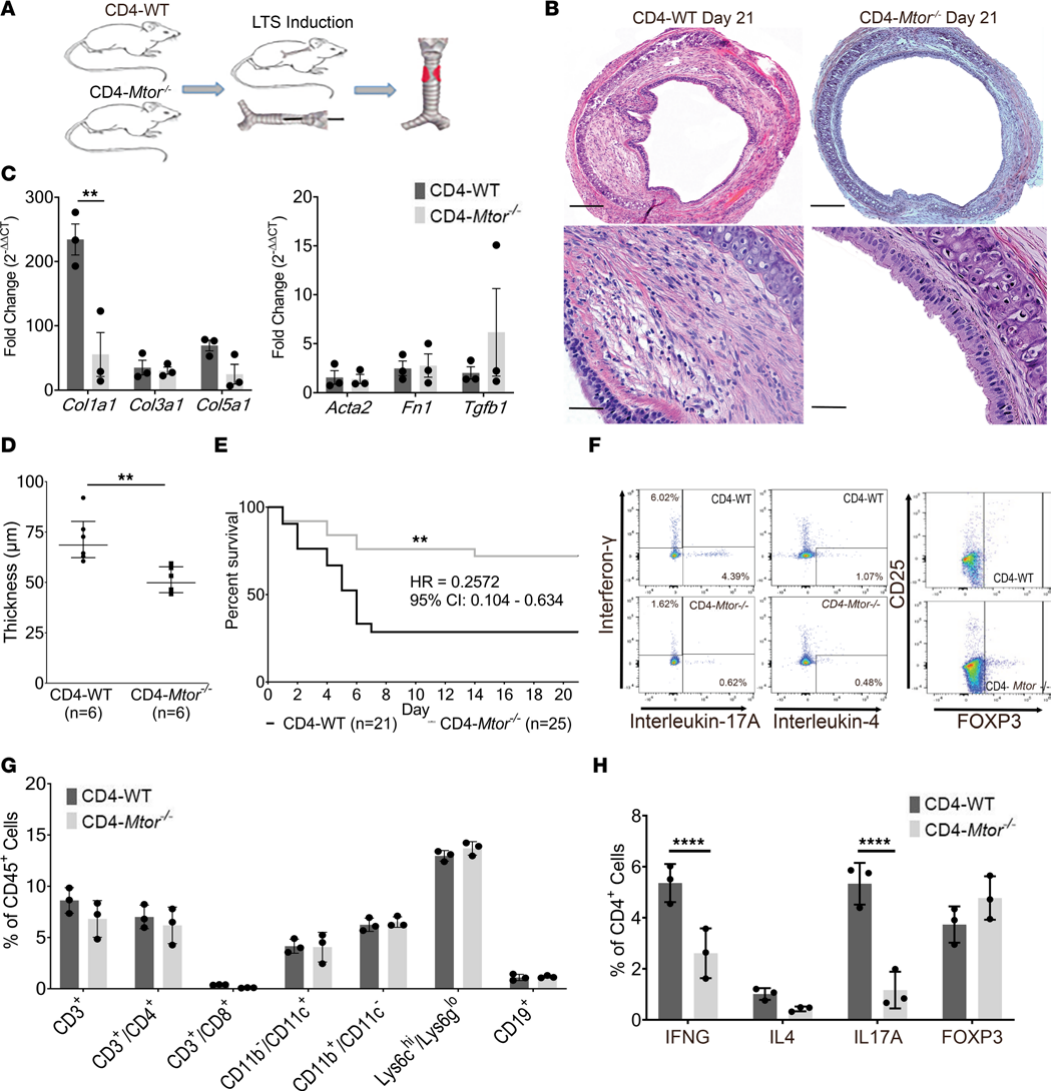
Selective deletion of mTOR in CD4 cells reduced fibrosis and improved survival in LTS mice. Bleomycin-induced LTS was assessed in CD4 Cre-*Mtor^fl/fl^* mice (CD4-*Mtor^–/–^*) and CD4 Cre-WT mice (CD4-WT) (**A**) Experimental design. (**B**) Representative histology (original magnification, ×10, top; ×40, bottom) of LTS-induced tracheas of day 21 CD4-*Mtor^–/–^* and CD4-WT mice. Scale bars: 200 μm (×10); 50 μm (×40). (**C**) Quantitative real-time PCR demonstrated reduced *Col1a1* expression (93.64 ± 70.28 vs. 232 ± 26.52) in CD4-*Mtor^–/–^* (*n* = 3) versus CD4-WT (*n* = 3) control mice at day 7. (**D**) Quantitative comparison revealed reduced tracheal lamina propria thicknesses at day 21 in LTS CD4-*Mtor^–/–^* (50.97 ± 2.677 μm, *n* = 6) compared with CD4-WT (71.48 ± 4.790 μm, *n* = 6) mice. (**E**) 21-day Kaplan-Meier curve demonstrated improved survival in CD4-*Mtor^–/–^* (gray line, *n* = 25) mice compared with CD4-WT (black line, *n* = 21) mice (HR = 3.887; 95% CI, 1.577–9.585; *P* < 0.01). (**F**) Representative flow cytometry plots of CD4^+^ T cell phenotypes from LTS-induced tracheas of CD4-*Mtor^–/–^* and CD4-WT mice. (**G**) Immune cell populations in LTS tracheas from CD4-*Mtor^–/–^* (*n* = 3) and CD4-WT (*n* = 3) mice at day 4. (**H**) Analysis of CD4^+^ T cell phenotype showed reduced Th1 (2.75 ± 0.709) and Th17 (4.167 ± 0.629) cells in CD4-*Mtor^–/–^* mice (*n* = 3) at day 4 after LTS induction. Flow cytometry data are presented as mean reduction ± SEM. A Mann-Whitney *U* test was used to compare LP thickness between CD4-*Mtor^–/–^* and CD4-WT mice and is presented as mean ± SEM. Survival differences were determined using a Mantel-Cox log-rank analysis and presented as a HR with 95% CI. A 2-way ANOVA was used to assess differences in immune cell populations. An unpaired *t* test comparing ΔΔCT values was used to assess changes in gene expression presented as average fold change (2^ΔΔCT^) ± SEM. **P < 0.01, ****P < 0.0001.

Using our bleomycin-induced model, we induced LTS in CD4 Cre-*Mtor^fl/fl^* (CD4-*Mtor^–/–^*) mice and CD4 Cre-WT control (CD4-WT) mice (Figure 3A). Compared with CD4-WT controls, CD4-*Mtor^–/–^* mice demonstrated minimal LP thickening on gross histologic examination of the tracheal LP at day 21 (Figure 3B). There was an associated reduction in *Col1a1* expression at day 7 (Figure 3C) in CD4-*Mtor^–/–^* mice, with nonsignificant changes at days 14 and 21 (Supplemental Figure 9). Histopathologic analysis showed lower LP thickness in CD4-*Mtor^–/–-^* mice compared with WT mice at days 7, 14, (Supplemental Figure 10), and 21 (50.9 ± 6.6 versus 71.5 ± 11.7 μm; *P* = 0.01) (Figure 3D), demonstrating CD4^+^ T cells’ role in propagating fibrosis in LTS. The antifibrotic effect observed in CD4-*Mtor^–/–^* mice was further demonstrated by increased survival in the 21-day Kaplan-Meier estimate of survival for the CD4-*Mtor^–/–^* mice (HR = 0.2572; 95% CI, 0.104–0.6343) (Figure 3E). IF for the myofibroblast marker ACTA2 revealed less staining in the LP of CD4-*Mtor^–/–^* mice compared with CD4-WT controls (Supplemental Figure 5, C and D).

Flow cytometry was then used to characterize the immune cell population and the CD4^+^ T cell phenotype (Figure 3F) in the tracheal LP of CD4-*Mtor^–/–^* and CD4-WT mice. LTS tracheas from CD4-WT and CD4-*Mtor^–/–^* mice revealed no differences in the total proportion of T cells, B cells, and macrophages (Figure 3G). However, a reduction in the number of CD4^+^ T cells expressing IFN-γ or IL-17A was observed in CD4-*Mtor^–/–^* compared with CD4-WT mice, indicating that selective deletion of *Mtor* in CD4^+^ T cells reduced Th1 and Th17 cell populations in LTS mice LP (*P* < 0.001 and *P* < 0.001, respectively, 2-way ANOVA) (Figure 3H). The regulatory T cell population was unchanged by selective deletion of mTOR in CD4^+^ T cells.

In WT LTS-induced mice, inhibition of IL-17A with a neutralizing antibody led to a reduction in LP thickness at day 21 when compared with mice treated with a vehicle control (28.33 ± 6.4 versus 72.7 ± 15.3 μm; *P* < 0.001) (Supplemental Figure 11, A and B). Additionally, IL-17A neutralization led to significant reduction in *Col1a1, Col3a1, and Col5a1* gene expression in LTS-induced tracheas (Supplemental Figure 11C). Expression of the myofibroblast marker *Acta2* (Supplemental Figure 11D) was also reduced in mice treated with an IL-17A–neutralizing antibody, and this reduction in α–smooth muscle actin was also demonstrated upon IF staining of the trachea LP (Supplemental Figure 11, E and F). α β

### Th17 CD4^+^ T cells promote collagen expression in human LTS fibroblasts.

**Figure 4:**
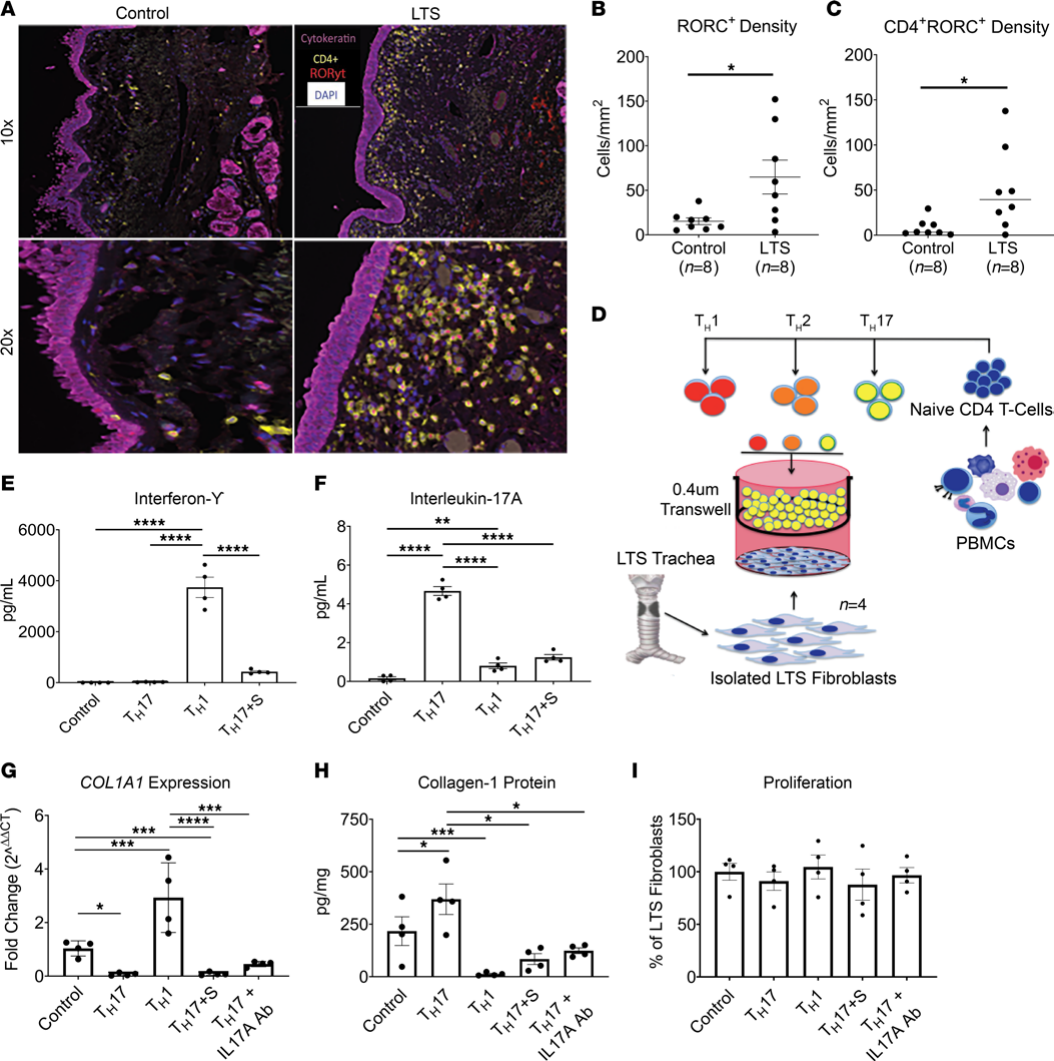
CD4^+^ T cell phenotype regulated profibrotic fibroblast function in vitro. (**A**) Representative images from multispectral IF analysis staining cytokeratin, CD4, RORγt (Th17 cell related transcription factor), and DAPI in normal controls (left) and human LTS specimens (right). Quantitative analysis of multispectral IF showed greater density of cells (percentage of total) expressing (**B**) RORγt (64.87 ± 18.99 versus 15.28 ± 3.77) and (**C**) colocalizing both CD4^+^ and RORγt (50.13 ± 16.29 versus 8.318 ± 3.40) in LTS (*n* = 8) compared with normal control specimens (*n* = 8). (**D**) Schematic of the in vitro coculture model that showed (**E**) skewed Th1 cells produced IFN-γ and (**F**) skewed Th17 cells produced IL-17A cytokines, respectively, with sirolimus inhibiting the production of IL-17A from Th17 cells. (**G**) Human LTS-derived fibroblasts increased *COL1A1* gene expression when cocultured with Th17 cells (3.360 ± 0.940); the increase was not present when Th17 CD4^+^ T cells were pretreated with sirolimus (Th17 ^+^ S) (0.089 ± 0.02) or when cultured with an IL-17A–neutralizing antibody (Th17 ^+^ IL-17A Ab) (1.39 ± 0.22). Human LTS-derived fibroblasts had lower *COL1A1* gene expression when cocultured with Th1 cells (0.055 ± 0.38). (**H**) Collagen-1 protein (pg/mg) was higher in human LTS-derived fibroblasts when cultured with Th17 cells (369.1 ± 72.72) than in control specimens (217.2 ± 68.51). This effect was not observed in Th17 ^+^ S (84.13 ± 25.87) or when treated with an IL-17A neutralizing antibody (Th17 ^+^ IL-17A Ab) (124.0 ± 13.62). (**I**) There was no difference in cell proliferation of LTS fibroblasts in coculture with Th1, Th17, Th17 ^+^ S, and Th17 ^+^ IL-17A Ab CD4^+^ T cells. Data are presented as mean ± SEM. A 2-way ANOVA was used to determine significant differences between coculture conditions, and a Bonferroni correction was used to account for multiple comparisons. Significant changes in gene expression were determined by comparing ΔCT values. Gene expression data are represented as average fold change (2ΔΔ^CT^) and SEM. *P < 0.05, **P < 0.01, ***P <0.001, ****P < 0.0001.

Given these findings, the influence of Th17 cells in human LTS was investigated. IF of human LTS specimens revealed an increased population of cells colocalizing CD4 and the Th17-associated transcription factor RAR-related orphan receptor C. When compared with normal control specimens, human LTS specimens demonstrated a greater density of cells expressing both CD4 and RAR-related orphan receptor C (50.13 versus 8.32 cells/mm^2^; *P* = 0.03) (Figure 4, A–C). To define effects of Th17 cells on LTS fibroblasts, the main effector cell in fibrosis, we used an in vitro coculture model (Figure 4D). In this model, LTS-derived human fibroblasts were cocultured with Th1 and Th17 CD4^+^ T cells generated from naive CD4^+^ T cells of healthy volunteers. Cytokine production was validated for each CD4^+^ T cell subtype, which revealed IFN-γ and IL-17A to be elevated in Th1 and Th17 cell culture, respectively (Figure 4, E and F). Subsequently, fibroblast collagen gene and protein expression was assessed after 72 hours in coculture in each condition. Th17 coculture resulted in increased *COL1A1* and collagen-1 protein expression relative to LTS fibroblasts cultured in isolation. The addition of an IL-17A neutralizing antibody to the culture conditions attenuated this profibrotic effect. Furthermore, the addition of sirolimus to the Th17 skewing conditions reduced IL-17A production (Figure 4F) and suppressed the increase in *COL1A1* and collagen-1 protein expression (Figure 4, G and H) while ACTA2 expression remained unchanged (Supplemental Figure 12). In contrast to Th17 coculture, LTS fibroblasts cocultured with Th1 cells had lower *COL1A1* and collagen-1 protein expression compared with control specimens. Fibroblast proliferation was not affected by CD4^+^ T cell phenotype, confirming that the increased collagen production was not a function of an increased number of fibroblasts when cocultured with Th17 cells (Figure 4I). These data indicate that Th17 cells elicit a profibrotic effect on LTS fibroblasts, which is abrogated when Th17 cells are treated with sirolimus.

### Local sirolimus delivery via a drug-eluting airway stent reduces fibrosis in LTS and suppresses Th17 infiltration.

**Figure 5:**
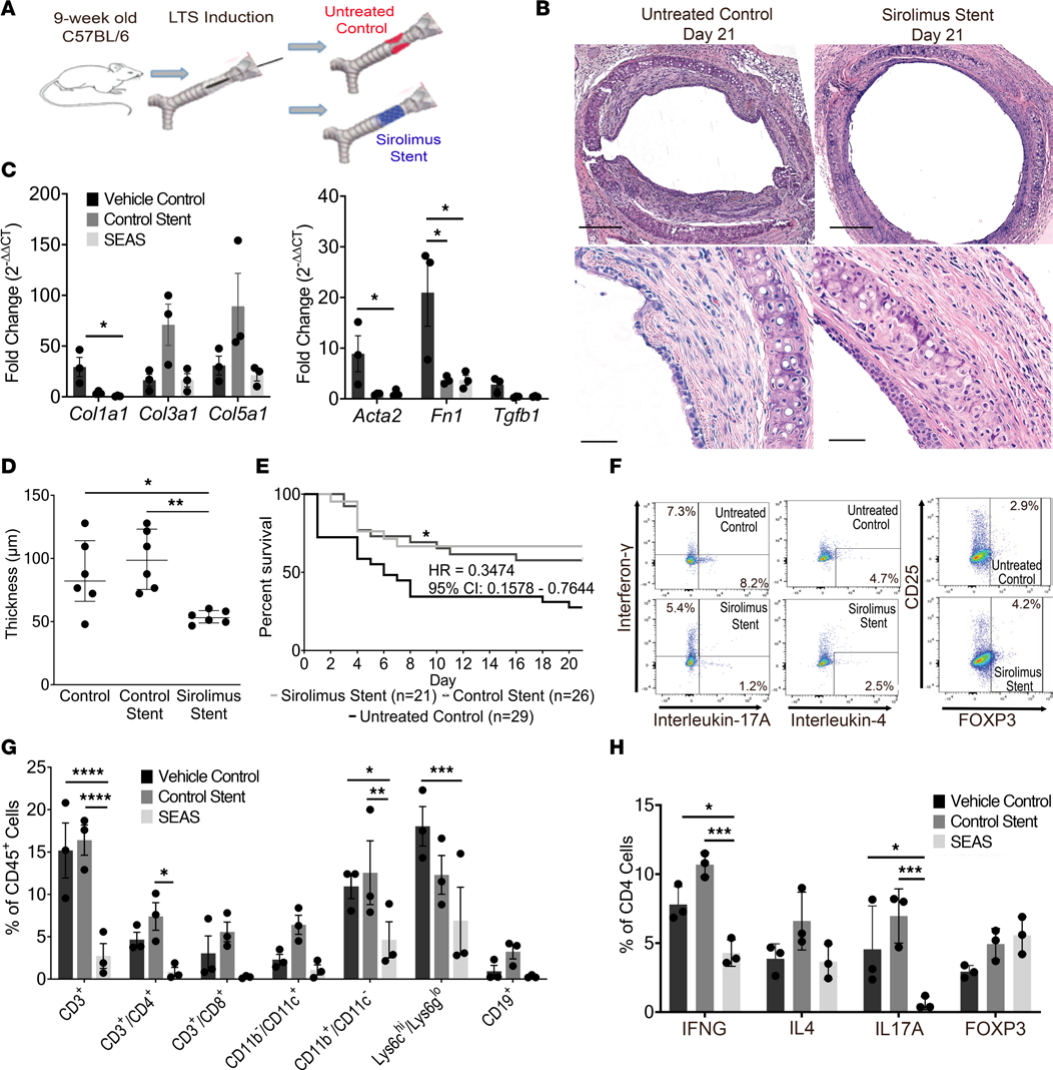
A sirolimus-eluting airway stent reduced fibrosis and improved survival in LTS mice. LTS-induced C57BL/6 mice were treated with a sirolimus-eluting airway stent (SEAS) or a control stent or were untreated. (**A**) Experimental design. (**B**) Representative tracheal histology (original magnification, ×10, top; ×40, bottom). Scale bars: 200 μm (10×); 50 μm (40×). (**C**) *Col1a1* (0.3382 ± 1.033 vs. 29.34 ± 9.515), *Acta2* (1.104 ± 0.3858 vs. 8.897 ± 3.561), and *Fn1* (3.676 ± 0.9896 vs. 20.96 ± 6.620) were reduced in LTS tracheas treated with SEAS (*n* = 3) compared with untreated controls (*n* = 3, day 7). (**D**) SEAS reduced tracheal lamina propria thickness at day 21 (53.52 ± 2.131 μm, *n* = 6) compared with control stent-treated mice (99.25 ± 9.674 μm, *n* = 6) and untreated controls (86.95 ± 11.60 μm, *n* = 6). (**E**) SEAS-treated mice (light gray line, *n* = 21) demonstrated improved survival compared with untreated controls (black line, *n* = 28) (HR = 0.3474; 95% CI, 0.1578–0.7644). Dark gray line indicates control stent treatment. (**F**) Flow cytometry plots of CD4^+^ T cell phenotypes. (**G**) LTS-induced tracheas (day 4) treated with SEAS (*n* = 3) demonstrated reduced CD3^+^ T (12.45 ± 3.562), CD11b^+^/CD11^–^ (6.310 ± 2.577), and Lys6c^hi^/Lys6g^lo^ (11.14 ± 4.587) cells when compared with untreated controls (*n* = 3) and reduced CD3^+^ (13.66 ± 2.303), CD4^+^ (6.523 ± 1.710), and CD11b^+^/CD11^–^ (7.917 ± 4.322) cells when compared with control stent-treated mice (*n* = 3). (**H**) SEAS (*n* = 3) reduced Th1 (3.520 ± 0.9323 and 6.410 ± 0.7470) and Th17 (3.910 ± 1.837 and 6.323 ± 1.167) cell populations in LTS tracheas when compared with untreated controls and control stent-treated mice, respectively. Flow cytometry data are presented as mean reduction ± SEM. A 2-way ANOVA was used to compare LP thickness between groups. Data are presented as mean ±SEM. Survival differences were determined using a Mantel-Cox log-rank analysis and presented as HR with 95% CI. A 2-way ANOVA was used to assess differences in immune cell populations. An unpaired *t* test comparing ΔΔCT values was used to assess gene expression changes presented as fold change (2^ΔΔCT^) ± SEM. *P < 0.05, **P < 0.01, ***P < 0.001, ****P < 0.0001. Bonferroni’s correction was utilized for multiple comparisons.

As an alternative approach to mitigate the potential risk of toxicity from systemic sirolimus, a biocompatible SEAS was developed and miniaturized for placement in mice (22) (Supplemental Figure 13). LTS mice treated with a SEAS were compared with mice receiving no treatment (Figure 5A) as well as a control stent (CS). Treatment with a SEAS resulted in less tracheal LP thickening on days 7, 14 (Supplemental Figure 14), and 21 (53.1 ± 5.2 versus 86.9 ± 28.4 μm; *P* = 0.041) (Figure 5, B and D). Additionally, *Col1a1* gene expression was reduced at day 7 when compared with untreated and CS-treated mice (*P* < 0.05) (Figure 5C). Finally, LTS mice treated with a SEAS demonstrated significantly greater survival than untreated mice (HR = 0.3474; 95% CI, 0.1578–0.7644; *P* = 0.008), but no difference in survival was observed between SEAS-treated mice and CS-treated mice (Figure 5E). ACTA2 expression, assessed by IF, was also visually reduced in the LP of SEAS-treated mice (Supplemental Figure 5, E and F). Systemic absorption of sirolimus from treatment with a SEAS was negligible (Supplemental Figure 15).

**Figure 6:**
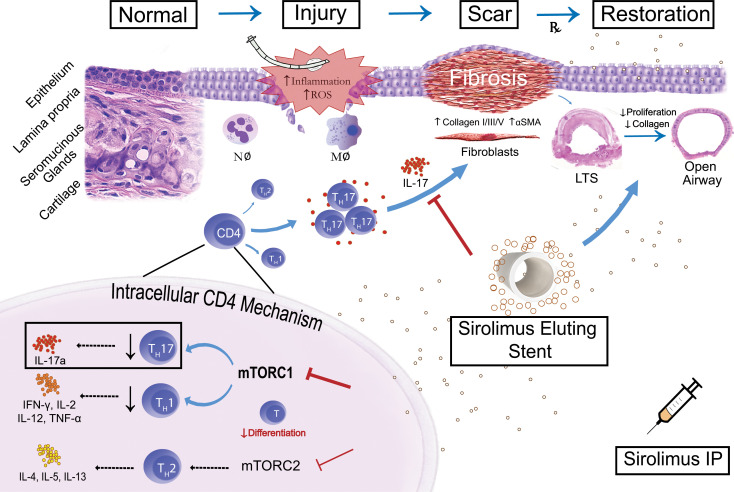
Schematic demonstrating the effect of mTOR inhibition on LTS. A visual schematic of how injury to normal tracheal epithelium by an endotracheal tube leads to excessive collagen and extracellular matrix deposition, hallmarks of LTS. Tissue injury and subsequent activation of local inflammatory mediators, including a prominent Th17 cell infiltrate, lead to pathologic LP fibrosis and LTS. Local and systemic mTOR inhibition with sirolimus reduces fibrosis and attenuates the development of LTS by reducing pathologic profibrotic Th17-mediated inflammation and by directly inhibiting fibroblast function.

Flow cytometry was performed to assess the immune cell population and CD4^+^ T cell phenotype (Figure 5F) in tracheas of mice treated with a SEAS as well as controls. Mice treated with a SEAS also demonstrated a global reduction in the CD45^+^ inflammatory cell population, similar to systemic sirolimus treatment (Figure 5G). Phenotypic analysis of the CD4^+^ T cell population from mice treated with the SEAS demonstrated relative reductions in the Th1 and Th17 populations compared with untreated controls and CS-treated mice (Figure 5H). Assessment of the CD8 T cell population revealed no difference in IL-17A expression in mice treated with systemic sirolimus or a SEAS compared with controls (Supplemental Figure 16). Furthermore, in human LTS, less than 5% of CD8 T cells expressed IL-17A (Supplemental Figure 17). In sum, local sirolimus delivery via a biocompatible SEAS attenuated fibrosis and reduced CD4^+^ Th17 cells with equivalence to systemic sirolimus (Figure 6).

## Discussion

Fibroproliferative disease is estimated to cause 45% of all deaths in the United States and represents the common endpoint of multiple diseases in different organ systems (24). Although the initial injury may be distinct, similar cellular and molecular dysfunctions causing unregulated CD4^+^ T cell–mediated inflammation, progressive fibrosis, and ultimately organ failure are seen in diseases such as heart failure, hepatitis, renal, and pulmonary fibrosis (24, 25). Our findings of a CD4^+^ Th17 cell infiltrate in human and murine LTS align it with the multiple fibrotic diseases in other organ systems (26–28). Furthermore, local delivery of the mTOR inhibitor sirolimus via a biocompatible drug-eluting airway stent was equivalent to systemic sirolimus in a murine LTS model. These findings depict mTOR’s critical role in the development of LTS fibrosis, identify mTOR as a therapeutic target, and validate a local drug delivery platform for management of LTS.

Aberrant mTOR signaling influences the development of fibrotic disease (19, 29, 30). mTOR is a central regulator of numerous cellular functions including metabolism, proliferation, and survival. In pulmonary and renal fibrosis, mTOR signaling pathways are upregulated in disease-related fibroblasts and promote collagen synthesis (30–32). The influence of mTOR signaling in CD4^+^ T cells on the development of fibrosis is less clear. In CD4^+^ T cells, mTOR signaling regulates the differentiation of Th1, Th2, and Th17 subsets through its subunit complexes mTORC1 and mTORC2 (17). Metabolic and environmental cues regulate these specific mTORC complexes in CD4^+^ T cells, yielding variable T cell activation and function (33). Commitment into Th1 and Th17 populations is dependent on activation of mTORC1 signaling, whereas Th2 cell differentiation requires mTORC2 activation (17, 34). Furthermore, mTOR inhibition has been reported to promote a regulatory T cell phenotype in vitro (34); however, this was not observed in our LTS model and may reflect the in vivo nature of our work. In fibroinflammatory disease, Th17 cells are implicated in promoting pathologic fibroblast activation and collagen deposition (26, 35, 36). In LTS, the population of CD4^+^ T cells in the fibrotic laryngotracheal LP is predominantly Th17. Given mTOR’s regulatory function in CD4^+^ T cell differentiation, as well as its role in fibroblast activation, mTOR inhibition presents as a viable strategy to attenuate fibrotic disease (19, 30, 31, 34).

In fibrosis, the role of Th1 and Th17 cells continues to be debated (37–39), with the role of each Th subset undefined in LTS. Although IL-17A signaling pathways are increased in LTS (14), a causal role for IL-17A signaling has not been demonstrated (14). In idiopathic pulmonary fibrosis, IL-17A signaling is upregulated and demonstrated to be pathologic in multiple models of lung fibrosis (27, 36, 40, 41). However, the cellular origin of pathologic IL-17A is unclear. In one report of pulmonary fibrosis, IL-17A–producing δγ T cells accentuate fibrosis independent of Th17 cells (29). Similarly, a study investigating human LTS attributed upregulation of the IL-17A signaling to IL-17–positive δγ T cells (14). Other reports, based on models of fibrotic disease across multiple organ systems, attribute pathogenesis to Th17-mediated inflammation (42, 43), whereas some acknowledge a role for both Th17 and δγ T cells (28). In vivo and in vitro data from this study suggest that Th17 cells and their associated cytokine IL-17A are responsible for the observed profibrotic effect in LTS.

Sirolimus is an FDA-approved mTOR inhibitor commonly used in renal transplant recipients to prevent allograft rejection and has demonstrated efficacy in treating autoimmune diseases (44–46). In LTS-induced mice, sirolimus attenuated fibrosis and resulted in global reduction of immune cells in the laryngotracheal LP, supporting its ongoing translation to clinical therapy for LTS. The observed antifibrotic effects in LTS-induced mice validate previous in vitro findings (47) and are consistent with sirolimus’s efficacy in idiopathic pulmonary fibrosis (18, 48, 49). Sirolimus produces an antifibrotic effect through direct inhibition of fibroblast activation. Additionally, we demonstrated that sirolimus’s immunosuppressive effect also drives the reduction in fibrosis, with reduced fibrosis in LTS-induced mice with a conditional deletion of mTOR in CD4^+^ T cells. These findings establish a clear role for CD4^+^ T cells in the development of LTS fibrosis and highlight sirolimus’s dual mechanism of action targeting both the pathologic CD4^+^ T cells and activated fibroblasts in LTS (50).

Given the potential for systemic side effects with systemic sirolimus, there is an advantage for local delivery of sirolimus. Previously, we designed and demonstrated proof of concept for a SEAS with a reliable drug release profile and mechanical stability (22, 51). In the current study, we rigorously tested the efficacy of the SEAS in LTS-induced mice and demonstrated a reduction in pathologic fibrosis equivalent to the observed effects of systemic sirolimus. Whereas the mechanical forces of the stent alone allow for increased airway patency, the SEAS provided distinct antifibrotic and antiinflammatory effects that mirror the treatment effects of systemic sirolimus, which were achieved without systemic absorption of the mTOR inhibitor. These findings indicate that local therapies are effective in treating LTS, suggesting LTS to be a locally mediated fibroinflammatory disease.

Targeting the fibroinflammatory infiltrate in LTS with local delivery of sirolimus is a potentially novel alternative treatment strategy for patients with LTS. In clinical medicine, stents are commonly used to treat obstructed anatomic conduits throughout the body, including arteries, ureters, bile ducts, large bronchial airways, and the trachea. Drug-eluting stents, such as sirolimus-eluting coronary artery stents, couple the mechanical properties of the stent with a locally delivered medication to prevent stenosis (52). In the trachea, mechanical stents are used to keep the airway patent in patients with malignant mediastinal tumors (53). However, in benign tracheal disease, such as LTS, stents are used less frequently because of complications with stent migration and patient intolerance when used over the long term (54). The placement of a short-term SEAS has the potential to transform current therapy for LTS by locally targeting pathologic T cells and fibroblasts to attenuate the development of fibrosis in the larynx and trachea.

There are limitations to our study. Human LTS demonstrates heterogeneity in its fibroinflammatory architecture and severity, making characterization of this disease challenging. Although it is heterogeneous, evidence of converging mechanisms promoting fibrosis is mounting, and similarities in the inflammatory cascade associated with pathologic fibrosis support common treatment modalities (55, 56). Other limitations stem from the application of our LTS animal model. In this study we used a murine model of acute LTS, which may not be fully reflective of human disease, a chronic condition with histology that shows acute on chronic inflammation (16). Therefore, the early mortality observed in our murine model was probably secondary to mucosal edema secondary to acute inflammation. Nevertheless, our model shows the development of fibrosis and can differentiate between treatment groups. Furthermore, common surgical treatments for LTS such as endoscopic excision and dilation, which are performed serially to increase airway patency, reinitiate the acute inflammatory cascade, similar to our murine model. Therefore, therapeutics that target acute inflammation (as seen in the mouse model) could be effective at attenuating human disease recurrence and promoting physiologic healing. Finally, although bleomycin is commonly used to incite fibrosis in multiple models, its effects may not fully recapitulate LTS pathology, inflammation, and disease progression. Additionally, bleomycin has been shown to lead to a strong inflammatory response (57) and can induce senescence in stromal cells, leading to a Th17 phenotype (58, 59). Therefore, the disruption of this accentuated inflammatory response with sirolimus may be what is driving the survival benefit in our murine model and therefore may not translate to the same benefit in human LTS disease.

In sum, mTOR’s central role in multiple pathogenic pathways contributing to fibrosis in LTS establish it as a rational therapeutic target (60). The mTOR inhibitor sirolimus targets both pathways and is an effective therapy for LTS that may be administered either systemically or locally via a drug-eluting airway stent. The preclinical efficacy of mTOR inhibition in LTS demonstrated here could shift the treatment paradigm and provides the rationale for a clinical trial.

## Methods

### Human tissue sampling and data collection.

Fresh LTS tissue specimens were obtained from patients undergoing tracheal resection for treatment of LTS. Normal control trachea specimens were collected from autopsy specimens as part of a rapid autopsy (<6 h) program. LTS and normal specimens were fixed in 10% formalin for 24 hours and mounted in paraffin. LTS and normal tracheal biopsy specimens were obtained at the time of direct laryngoscopy and bronchoscopy for management of LTS. Biopsy samples were processed, and genetic material was immediately extracted for generation of cDNA. *COL1A1* gene expression (F′-GAGGGCCAAGACGAAGACATC, R′-CAGATCACGTCATCGCACAAC) was analyzed by quantitative real-time PCR (qRT-PCR). LTS fibroblast cultures were generated from biopsy specimens of LTS scar obtained at the time of routine tracheal or subglottic dilation.

### Human histologic analysis and IF.

H&E-stained sections from 10 unique LTS specimens and 10 unique normal control trachea specimens were obtained. To quantify LP thickness, we split H&E-stained sections of the subglottis from LTS and normal control specimens into 5 equal segments. Using ImageJ software (NIH), we obtained measurements of the LP (medial edge of tracheal cartilage to epithelial basement membrane) in each segment and determined an average thickness for each specimen.

IF for CD4 and p-mTOR was performed with mouse anti-human CD4 (lot ab846; Abcam) and rabbit anti-human p-mTOR (Ser2448) (lot ab2855; Cell Signaling Technology). For each specimen, 4 areas of the highest-density staining were selected. A blinded pathologist independently performed quantification of CD4^+^ and p-mTOR^+^ staining cells. At a magnification of ×400, positive-staining cells were recorded.

Multiplex IF was performed for the following markers: CD4 (EP204, Sigma), FOXP3 (236A/E7, Abcam), and cytokeratin (AE1/AE3; Agilent) as previously described (61, 62) for human LTS specimens. Briefly, paraffin was removed from slides by heating, followed by antigen retrieval with ER2 (Leica Biosystems). Blocking/Ab diluent (Akoya Biosciences) was then used to prevent nonspecific staining. The first primary antibody was then applied. The position 1 polymer was then added, followed by the signal amplification dye (Opal Automation Multiplex IHC Kit, Akoya) for that antibody position. Antibody stripping, washing, and blocking were performed, and the sequence was repeated to amplify all 3 antibodies on the same slide. DAPI (Automation Multiplex IHC Kit, Ayoka) was applied and slides were mounted (ProLong Diamond, Thermo Fisher Scientific).

To quantify cell staining density, slides were scanned with a Vectra Polaris Quantitative Pathology Imaging System (PerkinElmer). Ten representative high-power images containing the submucosal interface and areas of intense inflammation were selected for subsequent analysis. Spectral unmixing, cell segmentation, and determination and quantification of cellular subsets were performed with InForm 2.4 Image Analysis software (PerkinElmer).

### Induction of LTS surgery.

Nine-week-old male and female C57BL/6 mice (Charles River Laboratories), CD4 Cre-WT, or CD4 Cre-*Mtor^fl/fl^* were used. Homozygous CD4 cre-*Mtor^fl/fl^* mice (strain 011009) and CD4 Cre (strain 022071) WT control mice on a C57BL/6 background (The Jackson Laboratory) were used for this study. Mice were anesthetized with an i.p. injection of ketamine at a concentration of 100 mg/kg and xylazine at a concentration of 10 mg/kg. The skin was sterilized with ethanol, a 1.5 cm vertical midline ventral incision was made over the laryngotracheal complex, and the larynx and trachea were exposed. A 22-gauge angiocatheter was used to transorally intubate the mice. Tracheal intubation (versus esophageal placement) was confirmed by direct visualization. Using a Seldinger technique, we used a 0.22 μm bleomycin-coated wire-brush to create a circumferential subepithelial tracheal injury to induce LTS (23). Postoperatively, mice were monitored until ambulatory. After surgery mice were maintained on a high-protein soft diet (ClearH_2_O). After 4, 7, 14, and 21 days mice were sacrificed, and their proximal trachea was removed. Samples for RNA isolation were stored at –80°C until extraction.

For studies investigating the use of i.p. sirolimus, C57BL/6 mice were randomly assigned to receive an i.p. injection of sirolimus (4 mg/kg) or a vehicle control before induction of LTS, and the operator was blinded to this randomization. i.p. sirolimus treatments were administered at the time of surgery and every 48 hours after surgery. For studies involving genetically modified mice, the operator inducing LTS was blinded to the underlying phenotypes. In studies investigating the efficacy of the sirolimus-eluting stent, mice were randomly assigned before LTS induction. Immediately after LTS induction, blinding was removed and mice randomly assigned to receive the sirolimus-eluting stent group received that treatment. For studies involving the use of a drug-eluting stent, LTS was induced in C57BL/6 mice, and mice were randomly assigned to SEAS, CS, or no treatment. The operator was blinded to randomization and treatment (SEAS versus CS). For studies investigating IL-17A, IL-17A neutralizing antibody (AB_1645260, BD Biosciences) or an IgG1 isotype control (AB_395507, BD Biosciences) was given through i.p. injection to mice at a concentration of 0.375 mg/kg. This was administered at the time of LTS induction and every 48 hours after surgery.

### Histology and tracheal LP measurements.

Whole tracheas harvested from each subject were fixed in 10% formalin for 24 hours and subsequently embedded in paraffin blocks. Eight 5 μm–thick sections of trachea roughly 250 μm apart, cut in the axial plane, were obtained. These sections were stained with H&E. A blinded pathologist then identified the section with the largest degree of LP thickening. Images of this section at ×10 magnification were obtained. The thickness of the LP was measured in ImageJ software. For this measurement, each specimen was split into 5 equal segments, and the thickest portion of each segment was measured and recorded. An average LP thickness was calculated for each specimen. Measurements were performed from the medial aspect of the tracheal cartilage to the basement membrane of the epithelium. Assessment and measurements of all images were performed in a blinded fashion. IF for ACTA2 was performed with rabbit anti-mouse ACTA2 (lot ab5694; Abcam). For each specimen, 4 areas of the highest-density staining were selected.

### RT-PCR.

To obtain mRNA, we performed lysis on whole tissues by using TRIzol reagent (Thermo Fisher Scientific) and in vitro cell culture samples by using RLT Plus buffer (QIAGEN). RNA purification was performed with RNeasy Mini Kits (QIAGEN). All qRT-PCR was performed with SYBR Green Master Mix (Applied Biosystems) according to the manufacturer’s instructions. Briefly, 2 μg enriched mRNA was used to synthesize cDNA with an iScript cDNA Synthesis Kit (Bio-Rad Laboratories). The cDNA concentration was set to 50–100 ng/well in a total volume of 20 μL. The qRT-PCRs were performed on the StepOnePlus Real-Time PCR System (Thermo Fisher Scientific). Primer sequences are listed in Supplemental Table 1. For all samples, ACTB was used as the reference gene, and samples were compared with vehicle control-treated specimens, WT controls, or untreated samples depending on experiment.

### Survival analysis.

A 21-day survival analysis was performed comparing LTS-induced C57BL/6 mice treated with i.p. sirolimus to those treated with an i.p. vehicle control, CD4 Cre-WT mice to CD4 Cre-*Mtor^fl/fl^* mice, and LTS-induced C57BL/6 mice treated with a SEAS compared with mice that received no treatment. Deaths within the first 24 hours of LTS induction were attributed to perioperative complications, and those mice were excluded from the cohort.

### Flow cytometry.

Fresh murine trachea specimens were digested for 45 minutes at 37°C with Liberase TL (1.67 Wünsch units/mL) (Roche Diagnostics) and deoxyribonuclease I (0.2 mg/mL) (Roche Diagnostics) in RPMI 1640 medium (Gibco). The digested tissues were processed through 70 μm cell strainers (Thermo Fisher Scientific), rinsed with PBS + 0.05% BSA, and then washed twice with 1× PBS. The enriched single-cell suspension was washed and stained with the following antibody panels. For assessment of the global immune cell population, after viability stain, a panel consisting of CD45, CD3, CD4, CD8, CD11b, CD11c, Lys6c, Lys6g, CD19, and Fc BLock TruStain FcX (Supplemental Table 2) was assessed on an Attune NxT Flow Cytometer (Thermo Fisher Scientific). For CD4 subset analysis, a panel consisting of a LIVE/DEAD stain, CD45, CD3, CD4, CD8, CD19, CD25, γδ TCR, FOXP3, IL-4, IFN-γ, and IL-17A (Supplemental Table 3) was assessed. Intracellular cytokine staining (ICS) followed fixation and permeabilization with BD Cytofix/Cytoperm Kit (BD Biosciences). Data analysis was performed in FlowJo flow cytometry analysis software (TreeStar).

Biopsies from areas of human LTS were procured at the time of routine dilation in the operating room. Fresh LTS biopsy specimens were digested for 45 minutes at 37°C with Liberase TL (1.67 Wünsch units/mL) (Roche Diagnostics) and deoxyribonuclease I (0.2 mg/mL) (Roche Diagnostics) in RPMI 1640 medium (Gibco). The digested tissues were processed through 70 μm cell strainers (Thermo Fisher Scientific), rinsed with PBS ^+^ 0.05% BSA, and then washed twice with 1× PBS. The enriched single-cell suspension was washed and stained with the following antibody panel: CD45, CD3, CD4, γδ TCR, IL-17A, IFN-γ, IL-4, p-mTOR, and LIVE/DEAD (Supplemental Table 4). ICS followed fixation and permeabilization with BD Cytofix/Cytoperm Kit (BD Biosciences). The stained cell suspensions were then assessed on a Aurora flow cytometer (Cytek).

### Sirolimus-eluting stent construction and placement.

A 70:30 mixture of poly-L-lactide and polycaprolactone (Evonik Industries) was dissolved in dichloromethane (Sigma) with 20% glycerol (Fisher Scientific) and 1% sirolimus in a vacuum chamber. SEASs were cast around the outer surface of a 22-gauge angiocatheter to achieve a stent lumen of 0.9 mm. Casted stents were left to dry in a vacuum chamber for 24 hours. Stents were removed from the angiocatheter and trimmed to a length of 3 mm and marked with a solitary black line. Stents with a weight between 0.4 and 0.5 mg (4–5 μg sirolimus) were selected for use in our in vivo model to ensure consistency.

### Sirolimus blood concentrations.

Sirolimus blood concentrations for mice treated with i.p. sirolimus, a SEAS, and a vehicle control were determined by mass spectrometry. Whole blood was obtained from tail vein puncture at day 4 after LTS induction. For i.p. sirolimus–treated mice, whole blood was obtained on day 4, 24 hours after the last administration of i.p. sirolimus. Whole blood was stored at –80°C until processing.

### Differentiation of CD4^+^ T cell subsets and CD4-LTS fibroblast coculture.

Biopsy specimens from human LTS scar were processed, and fibroblasts were isolated and expanded as previously described (63). Fibroblasts were limited to passages 2–4 for all experiments in this study. Blood from normal healthy human volunteers was collected, and a Ficoll-Paque Plus (GE HealthCare, Sigma) density gradient separation was used to isolate PBMCs. Naive CD4^+^ T cells were isolated using a MACS Naive CD4^+^ T Isolation Kit (Miltenyi BioTec Inc.). Isolated naive CD4^+^ T cells were plated at a concentration of 50,000 cells per well and differentiated into CD4^+^ T cell subsets using Cell X-Vivo media (Thermo Fisher Scientific) supplemented with CD3/CD28 Dynabeads (Thermo Fisher Scientific) and phenotype-specific cytokines (Supplemental Table 5) for 6 days. Sirolimus was added to the skewing conditions of Th17 at a concentration of 50 nM to generate a Th17-sirolimus subset. IL-17A neutralizing antibody (1F3E3, Proteintech) or an IgG1 isotype control was add to cell culture medium at a concentration of 500 ng/mL. T cell phenotype was confirmed by soluble cytokine production.

Confluent LTS fibroblasts were trypsinized, and 100,000 cells were passaged into the lower chamber of a 12-well 0.4 μm polystyrene Transwell plate (Falcon by BD Biosciences). Differentiated CD4^+^ T cell phenotypes were plated separately at a concentration of 200,000 cells per well in the upper chamber of the transwell system. LTS fibroblasts were cocultured with each CD4^+^ T cell subset as well as independently. Fibroblasts and culture media were collected after 72 hours. Collagen gene expression, soluble collagen production, and proliferation were assessed as previously described (22).

### Statistics.

All analyses were performed in Prism software, version 8.3.1 (GraphPad). Statistical analyses were performed via 2-tailed Student’s *t* test for parametric data and a Mann-Whitney test for nonparametric data as indicated in the figure legends. *P* values were corrected for multiple comparisons via a Benjamini-Hochberg procedure. For analysis of survival, a Mantel-Cox (log-rank) analysis was performed. *P* < 0.05 is regarded as statistically significant.

### Study approval.

This study was approved by the Johns Hopkins University Institutional Review Board (NA_00078310). Informed consent was obtained from all participants in alignment with the Johns Hopkins University Institutional Review Board. All murine protocols in this study were performed in accordance with an approved Johns Hopkins University Animal Care and Use Committee protocol (MO12M354).

### Data availability.

All data are available in the main text or the Supplemental Material.

## Author contributions

ATH, KMM, JDP, AG, JHE, SC, YCL, and MRH conceptualized the study. ATH, KMM, AG, JDP, JHE, SC, YCL, MRH, RJD, and LC provided design and methods. ATH, KMM, MKM, IS, IAL, SC, YCL, LC, MD, and RJD provided investigation. ATH, JHE, JDP, and MRH acquired funding. ATH, KMM, IS, MKM, and IAL provided project administration. ATH provided supervision. ATH, KMM, and IAL recruited patients. ATH, KMM, IS, and IAL wrote the original draft of the manuscript. All authors reviewed the manuscript for intellectual content and approved the final version.

## Supplementary Material

Supplemental data

## Version 1 05/09/2023

In-Press Preview

## Version 2 06/08/2023

Electronic publication
