# Supplementary material for: Sirolimus-eluting airway stent reduces profibrotic Th17 cells and inhibits laryngotracheal stenosis
Source: JCI Insight. 2023 Jun 8;8(11):e158456. doi: 10.1172/jci.insight.158456 (PMC10393235; doi:10.1172/jci.insight.158456)
Supplement: Supplemental data [file jciinsight-8-158456-s253.pdf]

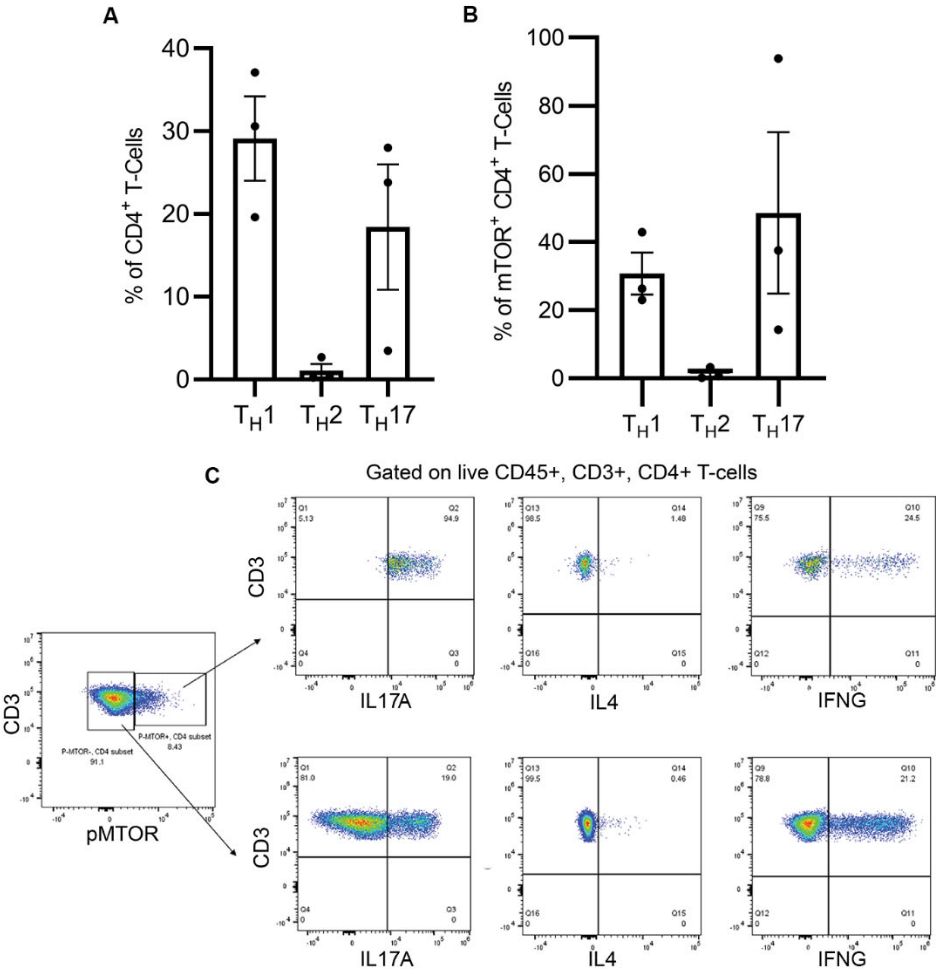

Fig S1. FACS analysis of human LTS biopsies. The proportion of CD4$^+$ T-cell phenotypes are presented for three human LTS specimens (A). The proportion of CD4$^+$ T-cell phenotypes presented for MTOR$^+$ CD4 T-cells (B). Representative gating strategy for CD4$^+$ T-cell phenotypes in human LTS (C).

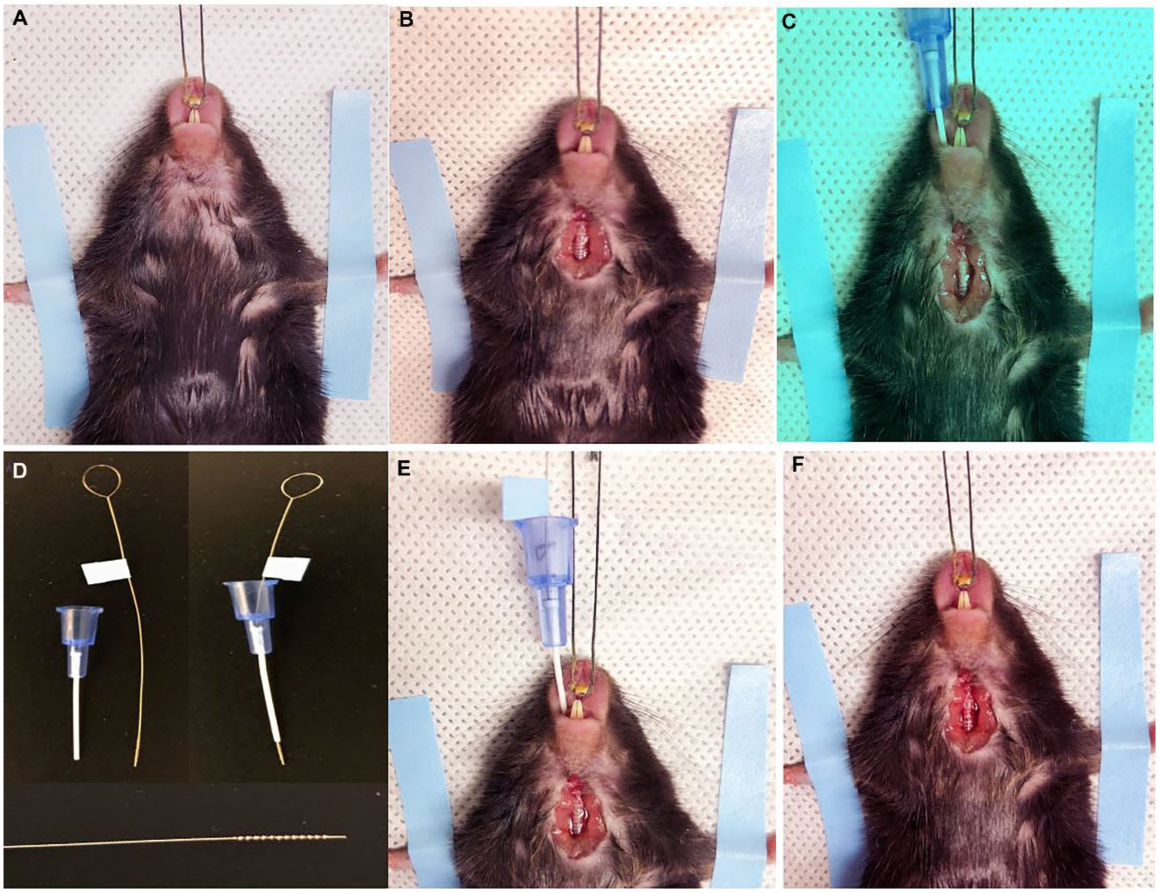

Fig S2. 9 week old mice were anesthetized with an intraperitoneal (IP) injection of ketamine (100 mg/kg), and xylazine (10 mg/kg). Mice were placed supine and suspended from the central incisors (A). After ethanol sterilization of the skin, a 1.5cm midline ventral vertical incision was made over the laryngotracheal complex (B) to expose the larynx and trachea. (C) A 22g angiocatheter was used to transorally intubate the murine trachea. Tracheal intubation (versus esophageal placement) was confirmed by direct visualization. A 0.22um wire brush that can be passed through the 22g angiocatheter was utilized (D). The wire-brush was coated in bleomycin (1U/mL) and using a seldinger technique (E), a circumferential endoluminal subepithelial tracheal injury was created (F) to induce laryngotracheal stenosis (LTS). After the injury is induced, the skin is approximated neatly with skin glue.

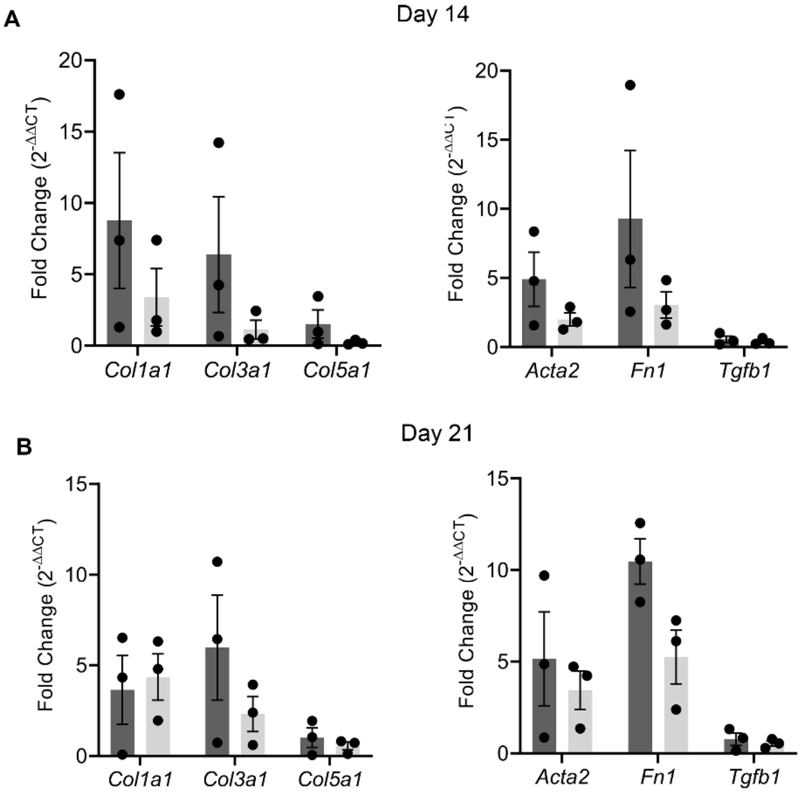

Fig S3. Fibrosis-related gene expression in LTS induced tracheas from control and IP sirolimus treated mice at day 14 and 21 (n=3) as determined by quantitative Real Time PCR analysis. An unpaired t-test comparing ΔCT values was used to assess changes in gene expression. Gene expression data is represented as average fold change ($2^{\Delta\Delta CT}$) and standard error.

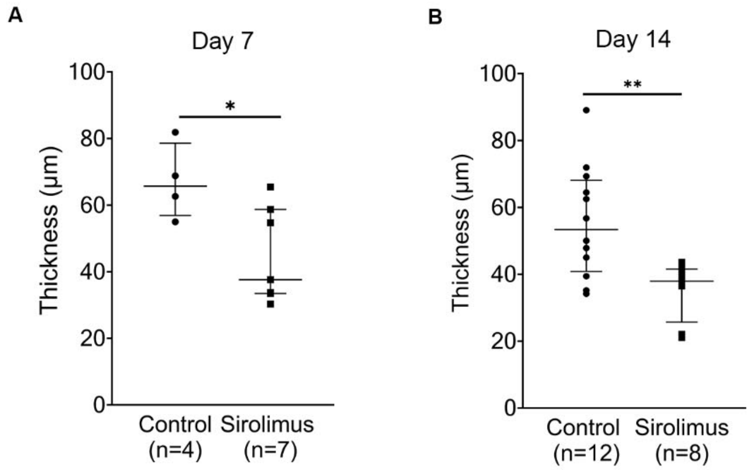

Fig S4. Histomorphometric comparison of tracheal lamina propria thicknesses (υm) at day 7 (A) and day 14 (B) in LTS induced C57BL-6 mice treated with IP sirolimus or control. Mann-Whitney U test was used for comparative analysis of LP thickness between IP sirolimus and vehicle control treated mice. Data is presented as mean and standard error. (*P<0.05, **P<0.01)

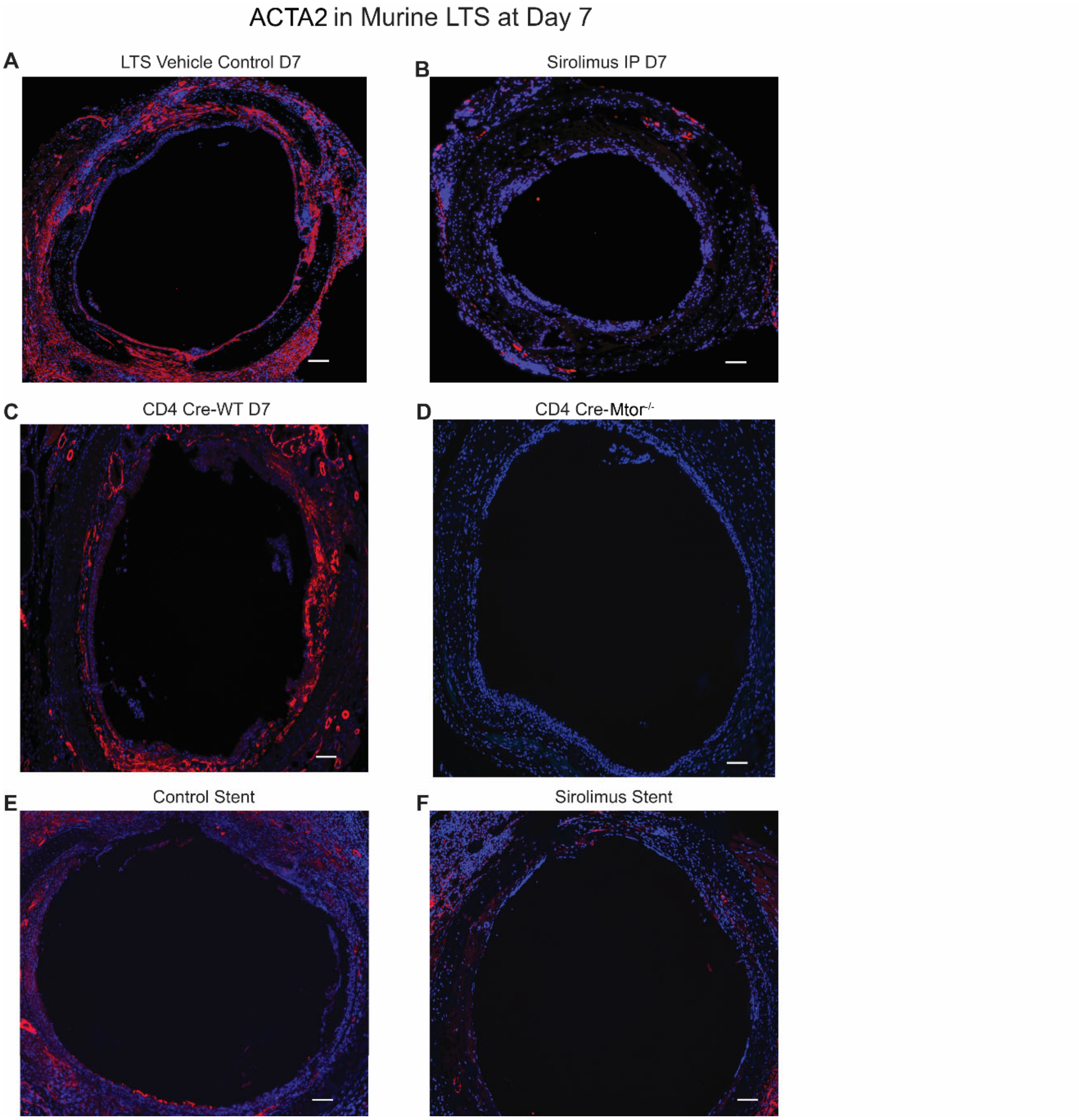

Fig S5. Immunofluorescence (10x) for the myofibroblast marker ACTA2 in murine LTS species at day 7 post LTS induction. (A) Vehicle control. (B) IP Sirolimus treatment. (C) CD4 Cre-WT mice. (D) CD4 Cre-*Mtor*$^{-/-}$ mice. (E) Control Airway Stent. (F) Sirolimus Eluting Airway Stent.

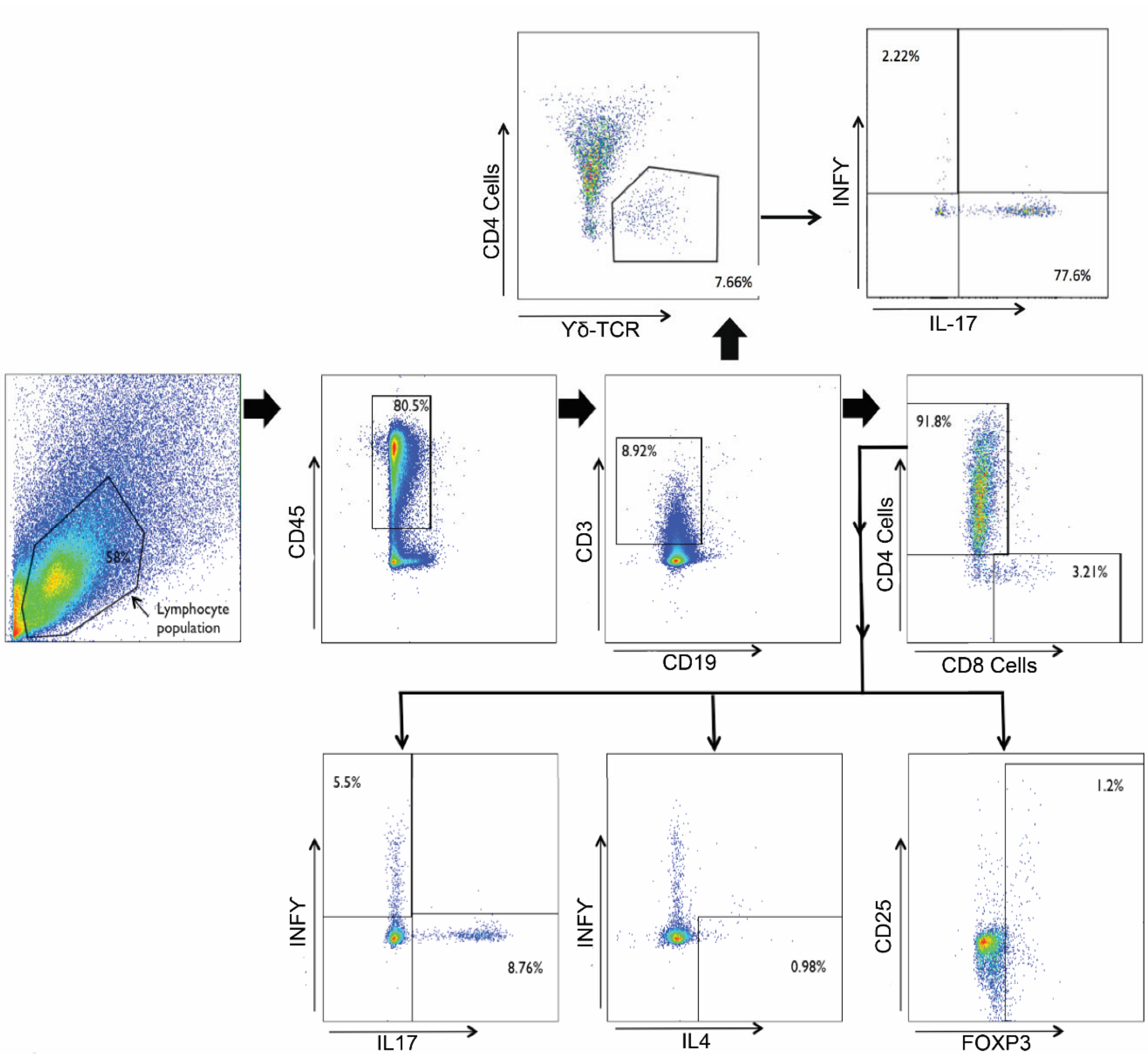

Fig S6. Depicted is the multicolor flow cytometry gating scheme used to determine the population of different CD4 T lymphocyte phenotypes and γδ T-cell populations in single cell suspensions from LTS murine trachea.

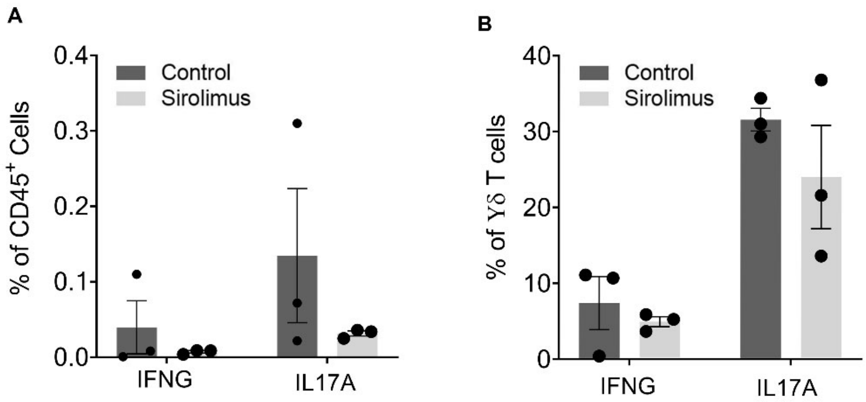

Fig S7. γδ T-cell populations in single cell suspensions from LTS induced tracheas of mice treated with IP sirolimus as determined by flow cytometry. (A) γδ T-cell populations as proportion of $CD45^+$ cells. (B) γδ T-cell populations as proportion of the total γδ T-cell population. Data is presented as mean and standard error. A 2-way ANOVA was used to determine significant differences in immune cell populations.

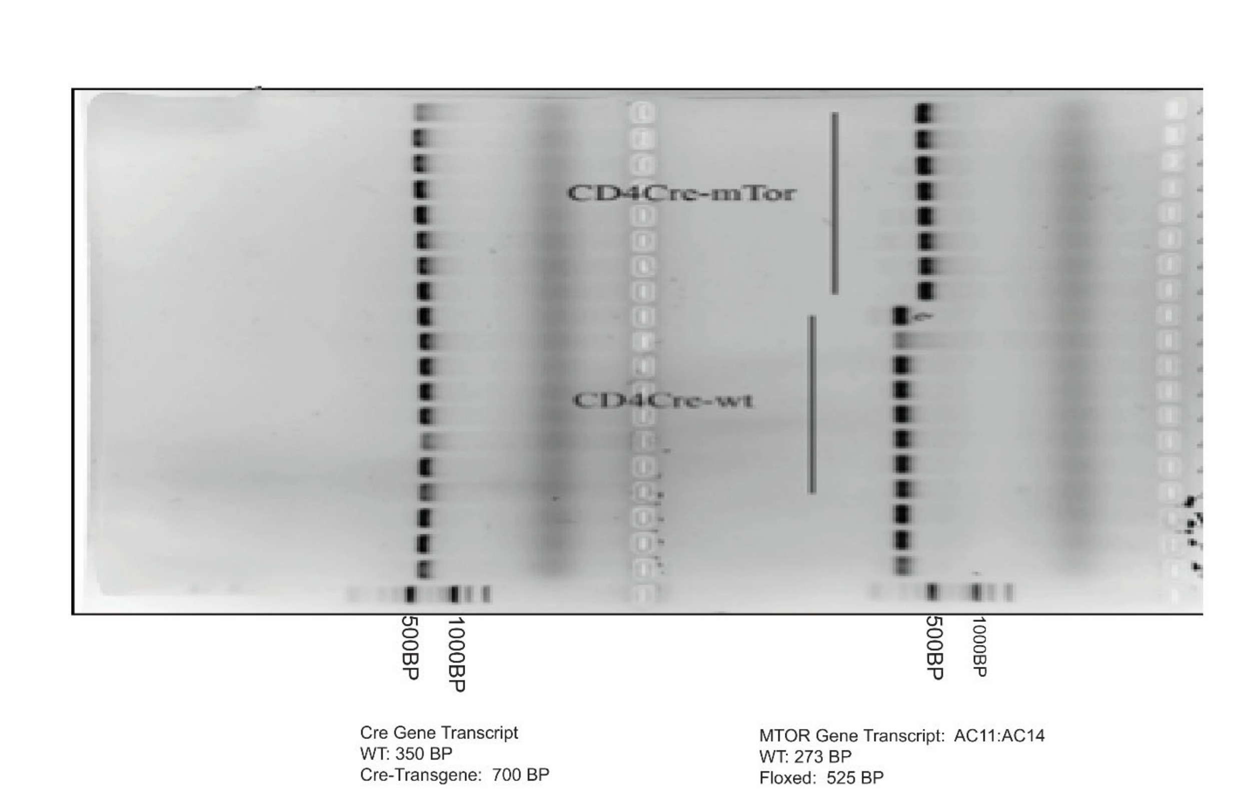

Fig S8. Confirmation of murine phenotypes by gel electrophoresis of PCR amplified genomic DNA constructs containing the CD4 cre transgene (left) and the floxed mTOR allele (525BP) or the WT *Mtor* allele (273BP) (right).

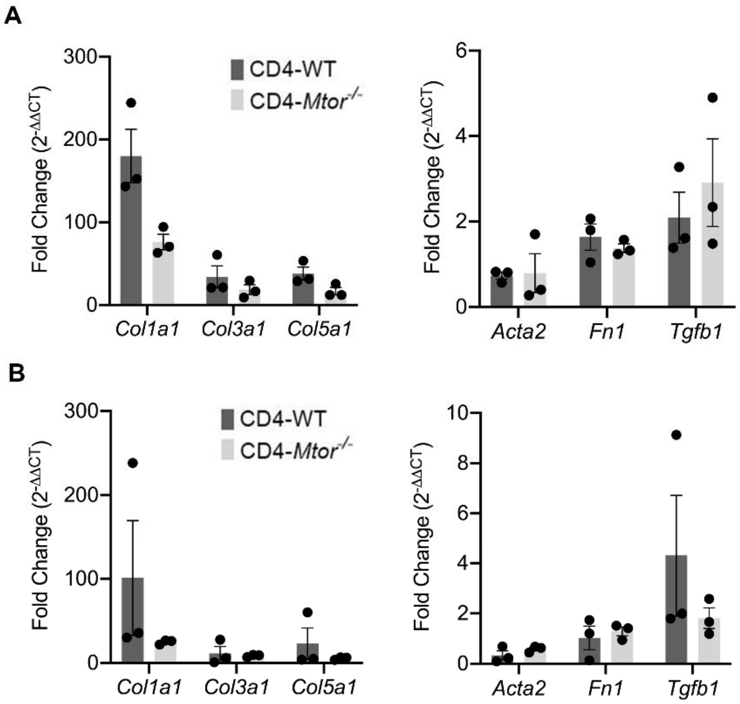

Fig S9. Fibrosis related gene expression in LTS induced tracheas from CD4 Cre-*Mtor*$^{-/-}$ and CD4 Cre-WT mice at day 14 and 21 (n=3) as determined by quantitative Real Time PCR analysis. An unpaired t-test comparing ΔCT values was used to assess changes in gene expression. Gene expression data is represented as average fold change ($2^{\Delta\Delta CT}$) and standard error. (*P<0.05, **P<0.01, ***P<0.001, ****P<0.0001)

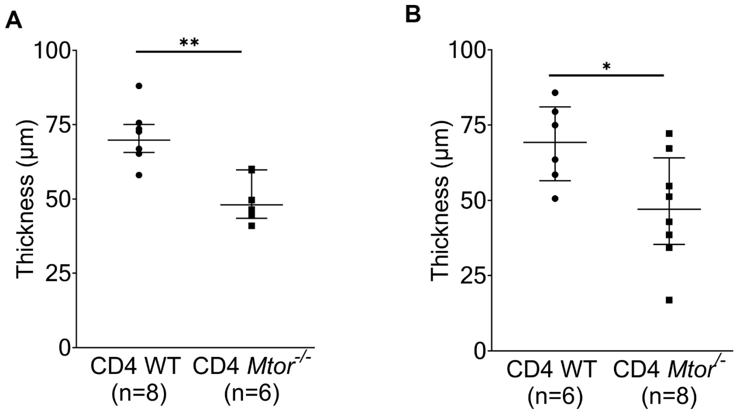

Fig S10. Histomorphometric comparison of tracheal lamina propria thicknesses (υm) at day 7 **(A)** and day 14 **(B)** in LTS induced CD4 Cre-*Mtor*$^{-/-}$ and CD4 Cre-WT mice. Mann-Whitney U test was used for comparative analysis of LP thickness between CD4 Cre- *Mtor*$^{-/-}$ and CD4 Cre-WT mice. Data is presented as mean and standard error. (*P<0.05, **P<0.01)

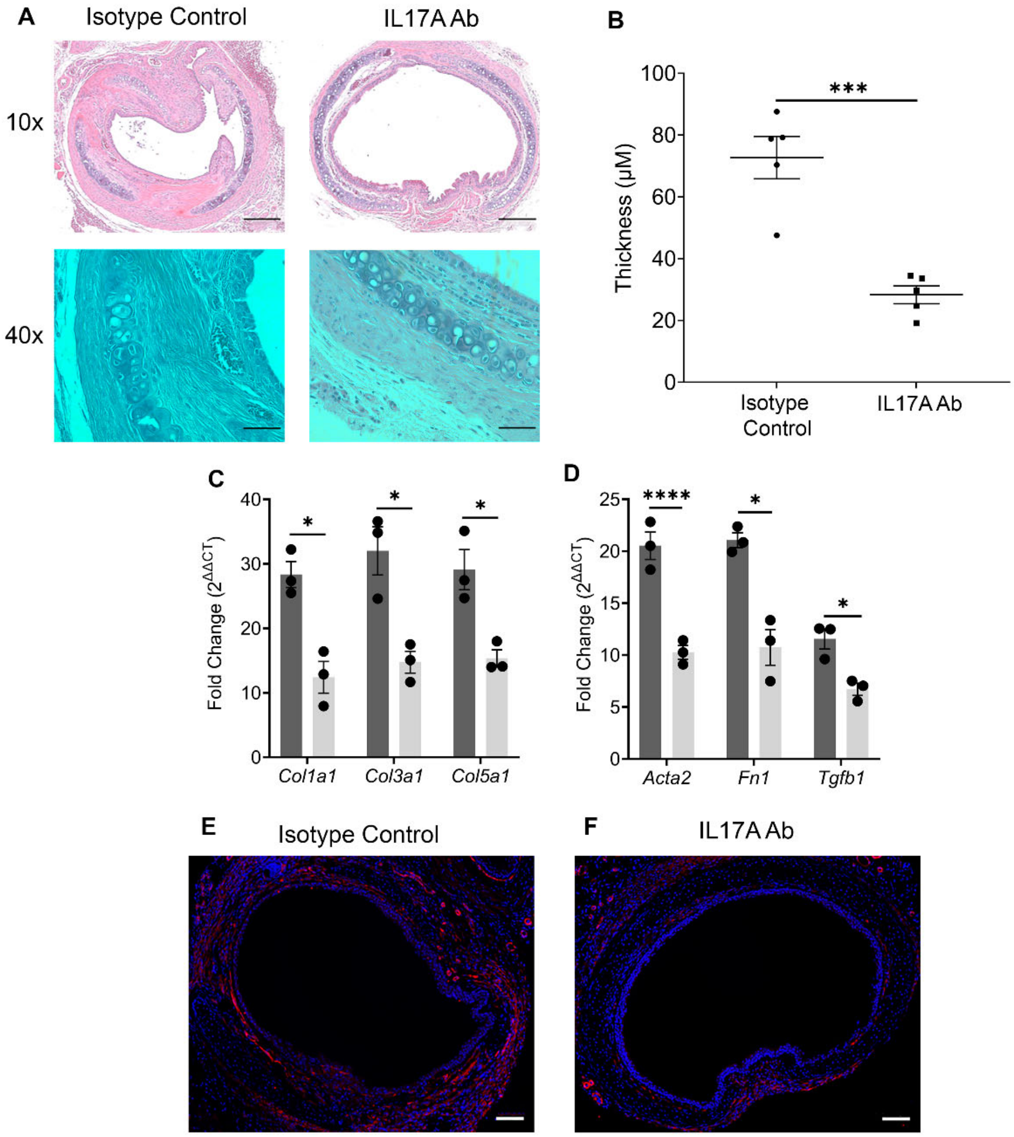

Fig S11. LTS was induced in C57BL/6 mice randomized to treatment with an IL17A neutralizing antibody or a vehicle control. **(A)** Representative histology (10x and 40x) at day 21 demonstrates LTS tracheas (black line represents lamina propria thickness) from mice following treatment with a IL17A neutralizing antibody or a vehicle control. **(B)** Histomorphometric comparison demonstrated reduced tracheal lamina propria thickness (μm) at day 21 in LTS mice treated with a SEAS (n=5) versus control (n=5). **(C)** Quantitative real time PCR analysis of fibrosis related gene expression revealed a reduction in *Col1a1*, *Col3a1*, *Col5a1*, *Acta2*, *Fn1*, and *Tgfb1* in LTS tracheas after treatment with a IL17A neutralizing antibody compared to mice treated with a vehicle control at day 7 (n=3), displayed as average fold change compared to healthy murine trachea from non-LTS induced C57BL/6 mice. Representative ACTA2 immunofluorescence in vehicle control (D) and IL17A neutralizing antibody (E) treated mice.

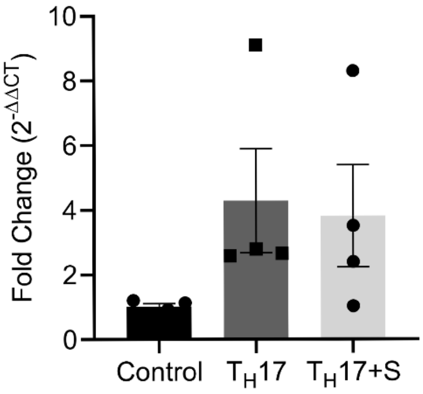

Fig S12. Gene expression of the myofibroblast marker alpha-smooth muscle actin (*ACTA2*) in fibroblasts isolated from LTS tissue co-cultured with T_H17 cells and T_H17 cells pre-treated with sirolimus (50nM) (T_H17+S). An unpaired t-test comparing ΔCT values was used to assess changes in gene expression. Gene expression data is represented as average fold change ($2^{\Delta\Delta CT}$) and standard error. (*P<0.05)

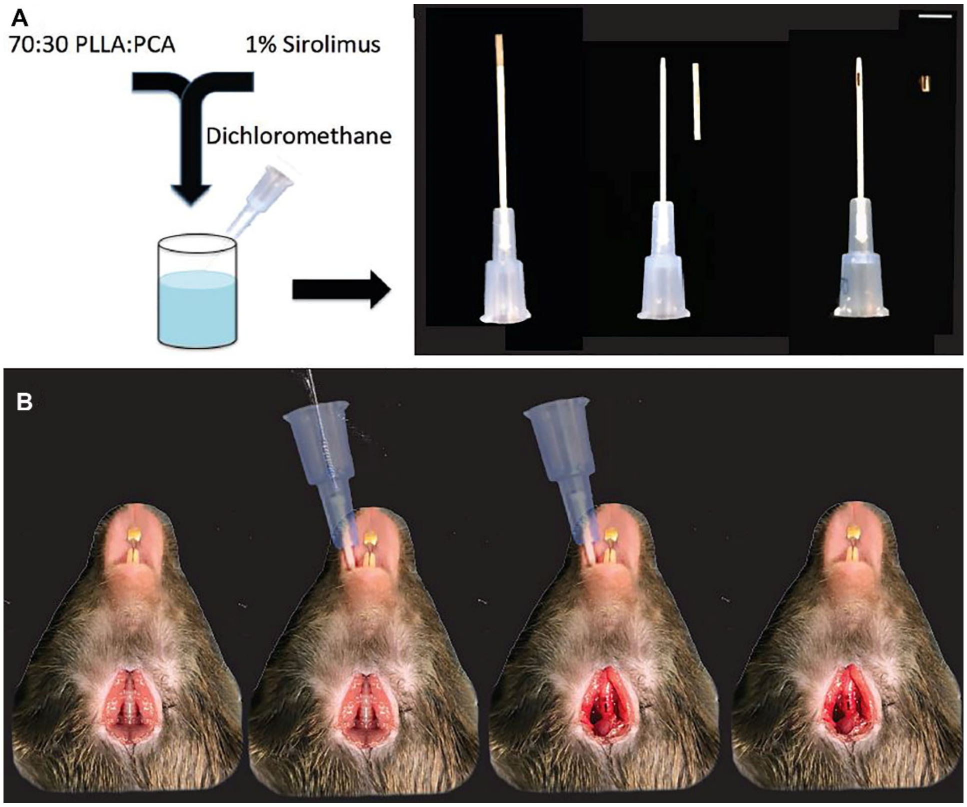

Fig S13. Sirolimus eluting airway stents were made from a 70:30 mixture of a PLLA:PCL with the addition 1% w/w of sirolimus dissolved in dichloromethane. The PLLA:PCL solution containing sirolimus was then cast around a 22g angiocatheter (A). The stents are allowed to dry in a fume hood for 24 hours and then the constructs are removed from the angiocatheter and cut into 3mm segments. Constructed stents were then loaded onto a fresh angiocatheter for placement. After the LTS injury is made, the sirolimus-eluting airway stent is placed using a seldinger technique (B). Placement was confirmed by visualization of the thin black marker on the stent through the translucent trachea.

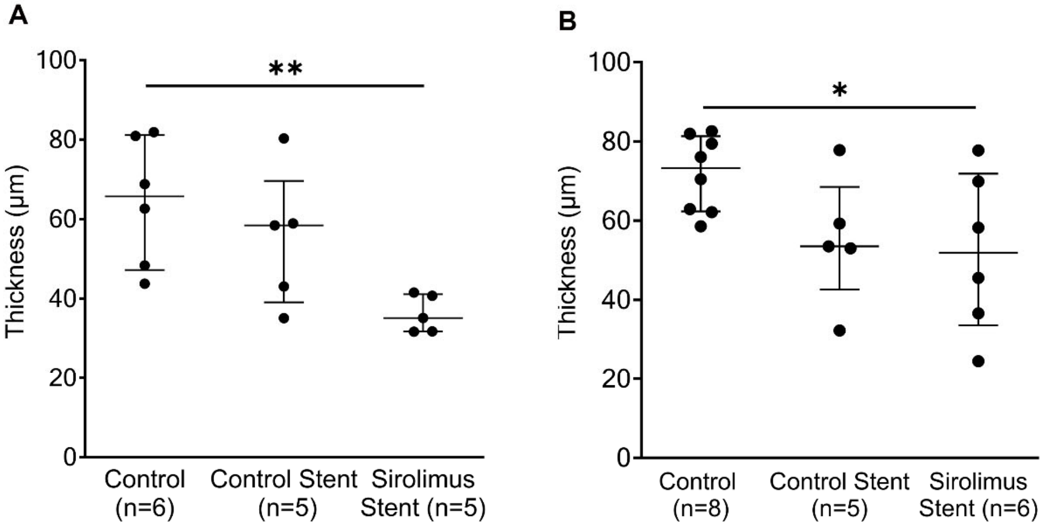

Fig S14. Histomorphometric comparison of tracheal lamina propria thicknesses (υm) at day 7 (A) and day 14 (B) in LTS induced C57BL-6 mice treated with a Sirolimus-eluting airway stent or or untreated controls. Mann-Whitney U test was used for comparative analysis of LP thickness between LTS mice treated with a sirolimus-eluting stent and untreated control mice. Data is presented as mean and standard error. (*P<0.05, **P<0.01)

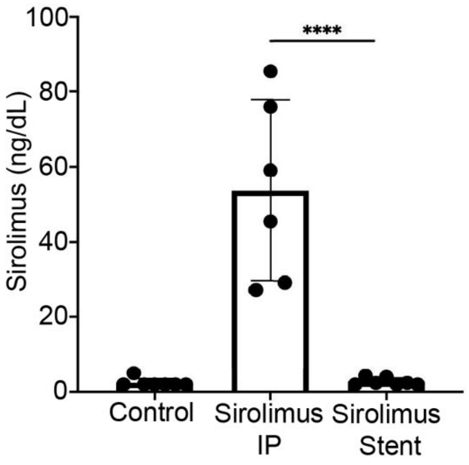

Fig S15. To assess for systemic absorption, sirolimus concentrations were assessed in whole blood obtained from mice treated with systemic sirolimus, a sirolimus-eluting airway stent, and a vehicle control. Sirolimus concentrations in whole blood were determined by mass spectrometry. A one-way ANOVA was used for comparative analysis of sirolimus blood concentrations in LTS mice treated with systemic sirolimus, a sirolimus-eluting airway stent, and a vehicle control. Data is presented as mean and standard error. (****P<0.0001)

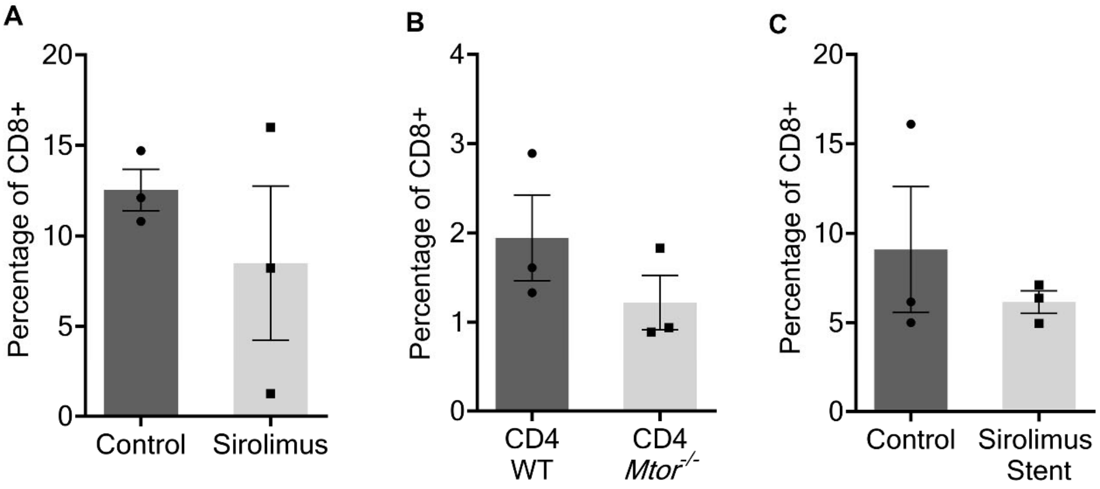

Fig S16. FACS analysis of the CD8 population demonstrating the proportion of $CD8^{+}$ T-cells localizing IL17A in control and IP sirolimus treated LTS mice (A), CD4 WT and CD4 *Mtor*$^{-/-}$ mice (B), and control and sirolimus eluting airway stent treated LTS mice (C).

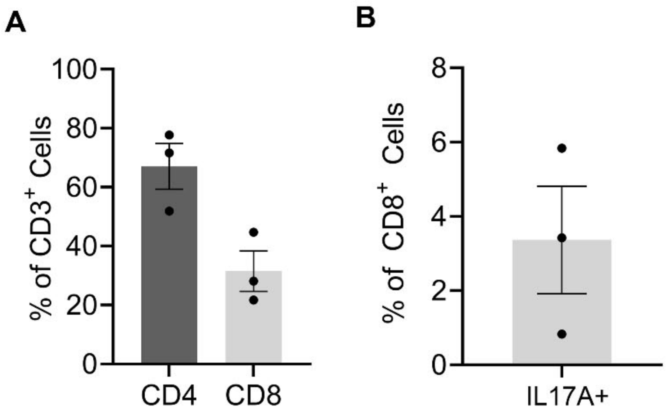

Fig S17. FACS analysis of T-cell populations (A) in biopsies of human LTS specimens (n=3). Proportion of $CD8^+$ T-cells that express IL17A in human LTS (B).

Table S1. Murine primer sequences

Gene	Forward Primer	Reverse Primer
Col1a1	5'-CTGCGGTTCAGGTCCAAT-3'	5'-TTCCAGGCAATCCACGAGC-3'
Col3a1	5'-CTGTAACATGGAAACTGGGGAAA-3'	%-CCATAGCTGAACTGAAAACCACC-3'
Col5a1	5'-AAGCGTGGGAAACTGCTCTCCTAT-3'	5'-AGCAGTTGTAGGTGACGTTCTGGT-3'
ACTA2	5'-CCCAGACATCAGGGAGTAATGG-3'	5'-TCTATCGGATACTTCAGCGTCA-3'
FN1	5'-GGACGCCAATTTGGAGGCT-3'	5'-CTTTCAGCGCATCGTGTCCT-3'
TGFB1	5'-GTTCTTCAATACGTCAGACATTCG-3'	5'-GTAACGCCAAGGAATTGTTGCTA-3'

Table S2: Immune Flow Cytometry Panel (murine)

Fluorophore	Marker	Dilution	Manufacturer (Catalog)
eFluor780	Live/Dead	1:1000	Life Technologies (65086514)
PerCP-Cy5.5	CD11c	1:250	BioLegend (337210)
PE	CD3	1:150	BioLegend (100205)
PE-Cy7	F4/80	1:150	BioLegend (123114)
PE-594	CD19	1:200	BioLegend (115554)
Pacific Blue	Ly6G	1:250	BioLegend (127612)
APC	CD4	1:250	BioLegend (116014)
AF700	CD11b	1:250	BioLegend (301356)
BV510	Ly6C	1:250	BioLegend (128033)
BV605	CD45	1:100	BioLegend (103139)
BV711	CD8	1:200	BioLegend (344733)

Table S3: CD4^{+} T-cell Subtype Panel (murine)

Fluorophore	Marker	Dilution	Manufacturer (Catalog)
Aqua	Live/Dead	1:1000	ThermoFisher Scientific (L34957)
FITC	Foxp3	1:200	BioLegend (320105)
PerCP-Cy5.5	CD19	1:100	BioLegend (152406)
PE-Cy7	CD4	1:150	BioLegend (100422)
PE-Texas Red	TCR γδ	1:100	BioLegend (331210)
PE	CD25	1:200	BioLegend (113704)
APC	IFNG	1:200	BioLegend (505810)
AF700	IL17	1:100	BioLegend (560613)
APC-eFluor780	CD3	1:200	ThermoFisher (47003282)
BV711	CD8	1:200	BioLegend (12633)
BV605	CD45	1:100	BioLegend (103140)
BV421	IL4	1:150	BioLegend (504120)

Table S4: Human Flow Cytometry Panel

Fluorophore	Marker	Dilution	Manufacturer (Catalog)
BV785	CD45	1:50	Biolegend (304048)
BV750	CD3	1:50	Biolegend (344846)
BUV661	CD4	1:25	BD Biosciences (612962)
BUV496	Gamma Delta	1:25	BD Biosciences (750020)
BV510	IL17A	1:25	Biolegend (512330)
BV711	IFN gamma	1:25	Biolegend (502540)
APC	IL4	1:25	Biolegend (500812)
FITC	Phospho-mTOR	1:25	Invitrogen (MA5-37392)
Zombie NIR	Live Dead	1:1000	Biolegend (423106)

Table S5: In-vitro CD4$^+$ T-cell Skewing Conditions

Cytokine	Company (Catalog)	T_H1 Conditions	T_H17 Conditions	T_H17+S Conditions
IL2	Peprotech (200-02)	5 ng/mL	--	--
IL12	Peprotech (200-12)	20 ng/mL	--	--
IL6	Peprotech (200-06)	--	30 ng/mL	30 ng/mL
IL23	Peprotech (200-23)	--	5 ng/mL	5 ng/mL
TGFB1	Peprotech (100-21)	--	1 ng/mL	1 ng/mL
Anti-IL4	Biolegend (500837	1 ug/mL	1 ug/mL	1 ug/mL
Anti-IFNG	Biolegend (506532)	--	1 ug/mL	1 ug/mL
Sirolimus	Sigma (1612765)	--	--	50nm
